# Med-BERT: pretrained contextualized embeddings on large-scale structured electronic health records for disease prediction

**DOI:** 10.1038/s41746-021-00455-y

**Published:** 2021-05-20

**Authors:** Laila Rasmy, Yang Xiang, Ziqian Xie, Cui Tao, Degui Zhi

**Affiliations:** 1 School of Biomedical Informatics, University of Texas Health Science Center at Houston, Houston, TX USA; 2 Peng Cheng Laboratory, Shenzhen, China

**Keywords:** Health care, Disease prevention, Experimental models of disease

## Abstract

Deep learning (DL)-based predictive models from electronic health records (EHRs) deliver impressive performance in many clinical tasks. Large training cohorts, however, are often required by these models to achieve high accuracy, hindering the adoption of DL-based models in scenarios with limited training data. Recently, bidirectional encoder representations from transformers (BERT) and related models have achieved tremendous successes in the natural language processing domain. The pretraining of BERT on a very large training corpus generates contextualized embeddings that can boost the performance of models trained on smaller datasets. Inspired by BERT, we propose Med-BERT, which adapts the BERT framework originally developed for the text domain to the structured EHR domain. Med-BERT is a contextualized embedding model pretrained on a structured EHR dataset of 28,490,650 patients. Fine-tuning experiments showed that Med-BERT substantially improves the prediction accuracy, boosting the area under the receiver operating characteristics curve (AUC) by 1.21–6.14% in two disease prediction tasks from two clinical databases. In particular, pretrained Med-BERT obtains promising performances on tasks with small fine-tuning training sets and can boost the AUC by more than 20% or obtain an AUC as high as a model trained on a training set ten times larger, compared with deep learning models without Med-BERT. We believe that Med-BERT will benefit disease prediction studies with small local training datasets, reduce data collection expenses, and accelerate the pace of artificial intelligence aided healthcare.

## Introduction

Artificial intelligence (AI)-aided disease prediction has undergone considerable development in recent years^1–3^. At present, it can improve the precision of diagnosis, enable disease prevention by early warning, streamline clinical decision making, and reduce healthcare costs^4–7^. Powerful AI tools, advanced conventional machine learning^8–10^, and deep learning^11–14^ approaches also have been widely applied in clinical predictive modeling and have gained numerous successes. Given enough training samples, deep learning models can achieve comparable or even better performance than domain experts in the diagnosis of certain diseases^15–19^. One prerequisite of typical deep learning-based methods is the availability of large and high-quality annotated datasets, which are used to model the underlying complex semantics of the input domain as much as possible and to avoid under-fitting of model training^20,21^. Big EHR data, however, often are not accessible for numerous reasons, including the limited number of cases for new or rare conditions; difficulty in data cleaning and annotation, especially if collected from different sources; and governance issues that hinder the data acquisition^22^.

Transfer learning was developed to address the issue whereby some representations were first pretrained on large volumes of unannotated datasets and then further adapted to guide other tasks^23^. A recent trend in transfer learning is to use self-supervised learning over large general datasets to derive a general purpose pretrained model that captures the intrinsic structure of the data, which can be applied to a specific task with a specific dataset by fine-tuning. This pretraining fine-tuning paradigm has been proven to be extremely effective in natural language processing (NLP)^24–30^ and, recently, computer vision^31,32^. Bidirectional encoder representations from transformers (BERT) is one of the most popular models for handling sequential inputs, e.g., text, with numerous variations^29,33–39^. BERT has also been embraced by the clinical domain^33,34,40^. However, these models were pretrained on clinical text and are only for clinical NLP tasks.

Structured EHR, as a primary input source for disease prediction, offers rich and well-structured information that reflects the disease progression of each patient and is one of the most valuable resources for health data analysis^41,42^. Adapting the transfer learning framework to structured EHR is a natural idea based on the analogy between natural language text and EHR, i.e., both are sequential modalities for tokens from a large vocabulary. However, a one-to-one mapping between the elements of natural language and structured EHR is not available.

There is a growing literature on transfer learning for EHR. Some researchers directly repurpose internal layers of trained deep models (e.g., RNN) for an existing task to a new task^43^ but these transfer learning might be too tightly coupled with specific tasks and its generalizability has not been well established. For the pretraining style transfer learning, previous studies on structured EHR showed some successes^44,45^ but they mainly focused on static embeddings such as word2vec^24^ and GloVe^25^, which failed to capture deep context information.

In this work, we choose the BERT framework, including its architecture and its training methodology, for training models on large EHR data. Notably, other contextualized pretrained embedding frameworks from the NLP domain, such as ULMFiT^46^ and ELMo^26^, could also be tested in the EHR domain. However, we choose BERT in this work because it is widely adopted with proven success.

To the best of our knowledge, there are only two relevant studies in the literature of the clinical domain: BEHRT^47^ and G-BERT^48^. These models, however, have the following limitations. BEHRT aims to develop pretrained models to predict the existence of any medical codes in certain visits. It uses positional embeddings to distinguish different visits and adds an age layer to imply temporal orders. The authors’ definition of the area under the receiver operating characteristics (AUC), however, was a non-standard one, making it difficult to compare their results with previous studies. G-BERT applied a graph neural network (GNN) model to expand the context of each clinical code through ontologies and jointly trained the GNN and BERT embeddings. It modified the masked language model (Masked LM) pretraining task into domain-specific ones, including maximizing the gap between the existing and non-existing codes and using different types of codes to predict each other. However, G-BERT’s inputs are all single-visit samples, which are insufficient to capture long-term contextual information in EHR. In addition, the size of their pretraining dataset is not large, making it difficult to evaluate its full potential. Furthermore, neither BEHRT nor G-BERT uses disease prediction tasks as the evaluation of their pretrained model by fine-tuning.

To alleviate the aforementioned issues and to evaluate a pretrained contextualized embedding model specific to disease prediction, we designed Med-BERT, an adaption of the BERT methodology for the structured EHR modality. Med-BERT is trained on structured diagnosis data coded using the International Classification of Diseases (ICD) codes, unlike the original BERT and most of its variations that were trained on free text. Note that we can also include other types of codes such as medications and laboratory tests, and we leave its investigation as future work.

We compare Med-BERT with BEHRT and G-BERT in Table 1. Remarkably, Med-BERT has a much larger vocabulary and a much larger pretraining cohort than the other two models, which help to provide a reality check of EHR BERT-based models. The larger cohort size and longer visit sequences in Med-BERT’s pretraining set will greatly benefit the model in learning more comprehensive contextual semantics. We also believe that, by using a large and publicly accessible vocabulary, i.e., ICD-9 and ICD-10, and pretraining the model on a multi-institutional dataset (Cerner), Med-BERT will likely be easily deployable to different institutions and clinical scenarios. Further, among all these pretrained models, only Med-BERT has been successfully cross-tested by a fine-tuning task on an external data source (Truven).

**Table 1:** Comparison of Med-BERT with BEHRT and G-BERT from multiple perspectives.

Criteria	BEHRT	G-BERT	Med-BERT
Type of input code	Caliber code for diagnosis developed by a college in London	Selected ICD-9 code for diagnosis + ATC code for medication	ICD-9 + ICD-10 code for diagnosis
Vocabulary size	301	<4 K	82 K
Pretraining data source	CPRD (primary care data)^63^	MIMIC III (ICU data)^64^	Cerner Health Facts (general EHR)
Input structure	Code + visit + age embeddings	Code embeddings from ontology + visit embeddings	Code + visit + code serialization embeddings
Pretraining sample unit	Patient’s visit sequence	Single visit	Patient’s visit sequence
Total number of pretraining patients	1.6 M	20 K	20 M
Average number of visits for each patient for pretraining	Not reported but >5	<2	8
Pretraining task	Masked LM	Modified Masked LM	Masked LM + prediction of prolonged length of stay in hospital
Evaluation task	Diagnosis code prediction in different time windows	Medication code prediction	Disease predictions according to strict inclusion/exclusion criteria
Total number of patients in evaluation tasks	699 K, 391 K, and 342 K for different time windows	7 K	50 K, 20 K, and 20 K for three task cohorts

Similar to BEHRT and G-BERT, Med-BERT made several modifications to the overall BERT methodology to fit the EHR data modality. Med-BERT used code embeddings to represent each clinical code, visit embeddings to differentiate visits, and the transformer structure to capture the intercorrelations between codes. Within each visit, we defined serialization embeddings to denote the relative order of each code, whereas neither BEHRT nor G-BERT introduced code ordering within a visit. In addition, we designed a domain-specific pretraining task prediction of prolonged length of stay in hospital (Prolonged LOS), which is a popular clinical problem that requires contextual information modeling to evaluate the severity of a patient’s health condition according to the disease progression and requires no human annotation. We expect that the addition of this task can help the model to learn more clinical and more contextualized features for each visit sequence and facilitate certain tasks.

The usefulness of the pretrained Med-BERT was evaluated by fine-tuning on the following two disease prediction tasks: the prediction of heart failure among patients with diabetes (DHF) and the prediction of onset of pancreatic cancer (PaCa), using three patient cohorts from two different EHR databases, Cerner Health Facts^®^ and Truven Health MarketScan^®^. These tasks are different from the pretraining prediction tasks (Masked LM and Prolonged LOS) and, thus, are good evaluation tasks to test the generalizability of the pretrained model. In addition, we chose these tasks because they capture more complexity than merely the existence of certain diagnosis codes, and are based on established phenotyping algorithms that further integrate multiple pieces of information beyond diagnosis codes, such as constraints on time window, diagnosis occurrence times, medications, and laboratory test values.

Fine-tuning experiments were conducted for the following purposes: (1) to test the performance gains by adding Med-BERT on three state-of-the-art predictive models; (2) to compare Med-BERT with a pretrained non-contextualized embedding, the clinical word2vec-style embedding^45^; and (3) to see how much Med-BERT would contribute to disease predictions with different fine-tuning training sizes.

Our primary contributions are summarized as follows:

1. This work is the first proof-of-concept demonstration that a BERT-style model for structured EHR can deliver a meaningful performance boost in real-world-facing predictive modeling tasks.
2. We innovatively designed a domain-specific cross-visit pretraining task that is prevalent among EHR data and is effective in capturing contextual semantics.
3. This work is the first demonstration of significantly boosted performance over state-of-the-art methods on multiple clinical tasks with phenotyped cohorts.
4. This work is the first that presents the generalizability of EHR BERT models by boosting the performance in a dataset (Truven) other than the training dataset (Cerner).
5. The performance boost of Med-BERT is observed across all sample sizes, demonstrating the enabling power of pretrained models for clinical tasks for which only limited training data are available.
6. We provided a visualization tool to demonstrate the dependency semantics in EHRs, facilitating the interpretability of the model.
7. We made our pretrained models and code available, enabling its applications by other researchers.

## Results

### Data source

We extracted our cohorts from two databases: Cerner Health Facts^®^ (version 2017) (Cerner) and Truven Health MarketScan^®^ (Truven). Cerner is a de-identified EHR database that consists of over 600 hospitals and clinics in the United States. It represents over 68 million unique patients and includes longitudinal data from 2000 to 2017. The Truven Health MarketScan^®^ Research Database (version 2015) is a de-identified patient level claims dataset. It represents over 170 million patients from 2011 to 2015 from commercial insurance, Medicare supplemental claims, and Medicaid claims.

**Fig. 1:**
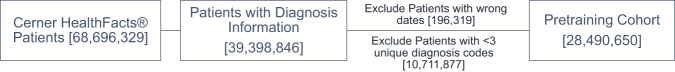
Selection pipeline for the pretraining cohort from Cerner HealthFacts. The flow starts from left to right. Number of patients is between square brackets.

Our pretraining cohort for Med-BERT is consisting of 28 million patients extracted from Cerner (Fig. 1). For model evaluation, we extracted three phenotyped cohorts, two of which were from Cerner (DHF-Cerner and PaCa-Cerner) and one from Truven (PaCa-Truven). The descriptive analysis of these cohorts is shown in Table 2, see “Methods”: Cohort definition for details.

**Table 2:** Descriptive analysis of the cohorts.

Characteristic	Pretraining	DHF-Cerner	PaCa-Cerner	PaCa-Truven
Cohort size (*n*)	28,490,650	672,647	29,405	42,721
Percent of patients with the event^a^	15%	14%	0.07%	0.06%
Average age on last/index encounter (std)	41	61	65	63
Gender—Male (%)	45%	47%	45%	48%
Race				
White (%)	68%	72%	77%	NA
African American (%)	15%	16%	13%	
Asian/Pacific Islander (%)	2%	2%	2%	
African American (%)	2%	2%	1%	
Average number of visits per patient	8	17	7	19
Average number of codes per patient	15	33	14	18
Vocabulary size	82,603	26,427	13,071	7002
ICD-10 codes (%)	33.8%	13.3%	20.7%	0%

^a^The event for pretraining is a prolonged hospitalization >7 days. The event for DHF-Cerner is the development of heart failure for diabetic patients. The event for PaCa-Cerner and PaCa-Truven is the diagnosis of pancreatic cancer and the percent is from the dataset total population.

### The data modality of structured EHR

We define structured EHR data of each patient as a sequence of visits, each as a list of codes. This is a classic formulation commonly used in the literature^12,49–51^. The codes within a visit can be either ordered or unordered. If unordered, the EHR data for each patient can be reduced to a sequence of sets. The Med-BERT framework can handle both ordered and unordered codes inside a visit. In this paper, we have access to the priority of the diagnosis codes as coded by billers, e.g., the primary diagnosis is mostly assigned the first priority followed by the second most important diagnosis and so on, and thus we encode that information to introduce order.

**Fig. 2:**
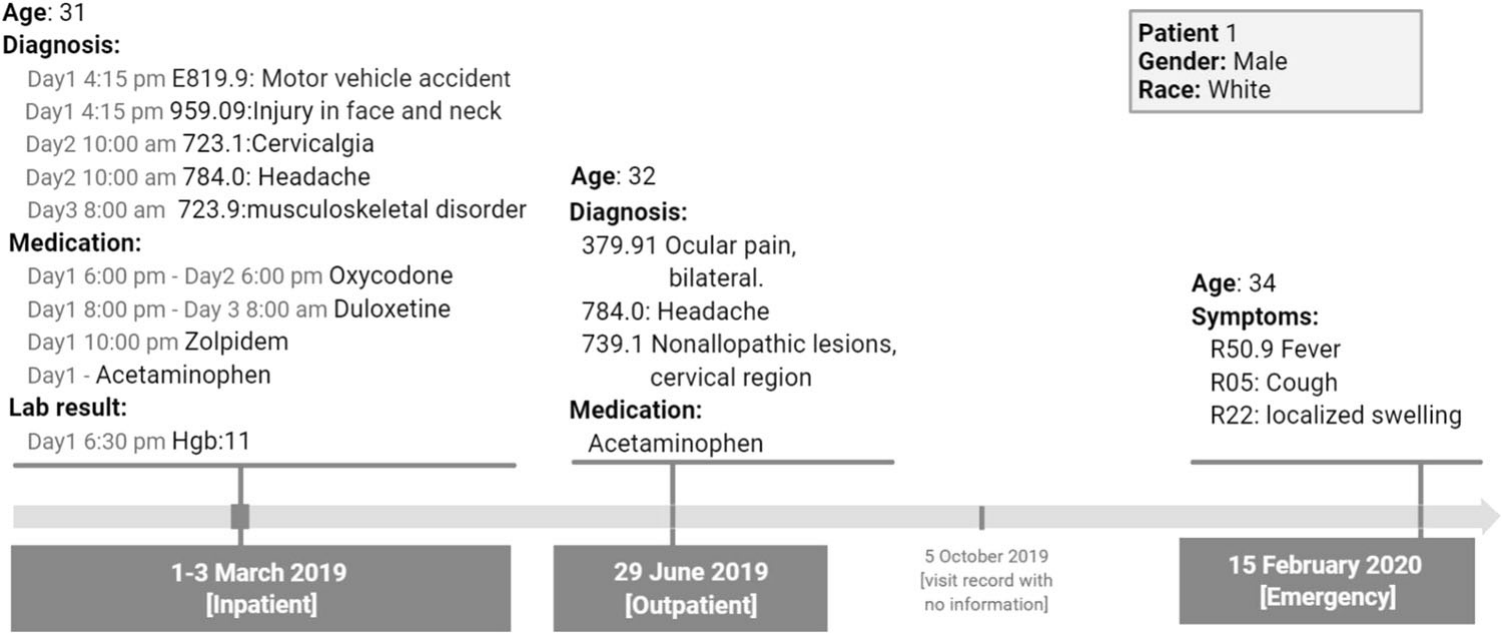
An example of structured EHR data of a hypothetical patient as it would be available from a typical EHR system (e.g., Cerner or Truven). For this patient, four visits with dates and encounter types are organized according to chronological order at the bottom. Detailed information including demographic and medical codes with time stamps are shown above. Note that not all information is recorded, as in real-world EHR recording system.

Both structured EHR and natural language text are sequential data with tokens. Therefore, the data modality of EHR are similar to text in many ways. However, EHR data have distinct characteristics (Fig. 2). A direct comparison between the data modalities of the structured EHR data with the natural language text is shown in Table 3.

**Table 3:** Comparison of characteristics of EHR data versus Natural language data.

Criteria	Natural language	EHR
Token granularity	The basic token is a word, which is a compressed semantic unit in language and can express some basic meaning. But in many cases, an integrated semantic unit (e.g., a named entity or a prepositional phrase) requires the combination of multiple tokens.	The basic token is a clinical code, which can represent an integrated semantic unit, e.g., a disease description, a drug, or a procedure.
Syntactic: Hierarchical structure	A paragraph (document) contains multiple sentences, and a sentence contains multiple words.	More complex, a patient’s information contains multiple visits, and a visit contains multiple codes of different categories.
Syntactic: Sequential order	Simple and clear.	The visits are sorted sequentially according to time but the codes within a visit may be unordered or with certain prioritized orders.
Semantic	Dependency relations among sentences (e.g., discourse relations) as well as words within each sentence (e.g., syntactic dependency, semantic roles) are clear.	Dependency relationships are not always clear, e.g., adjacent visits may be of little relevance owing to large time intervals.
Time interval	Regular, one between adjacent words.	Usually no explicit intervals between codes, and irregular intervals between adjacent visits.
Data completeness	Relatively complete for regular texts such as written language.	Usually incomplete and sometimes erroneous due to the nature of EHR.
Sequence length	Within a relatively narrow range: the maximum sequence length of words in a sentence rarely reaches a hundred.	More variable: a patient’s medical records can include anywhere from one to hundreds of visits. In a single visit, a patient can have hundreds of medical codes.

### Med-BERT architecture

In this work, we utilized essentially the same transformer architecture as that in the original BERT paper^29^, including multi-level embeddings and bidirectional transformers. We also adopted similar pretraining techniques (same loss function on masking and classification pretraining tasks). Still, given the semantic differences between EHR and text, adapting the BERT methodology to structured EHR is non-trivial. For example, while the input modality of the original BERT was a 1-D sequence of words, our input modality is structured EHR which is recorded in a multilayer and multi-relational style. There are no clear rules on how to flatten the structured EHR into a 1-D sequence and how to encode the “structures” of the structured EHR in the BERT transformer architecture. In addition, it is unclear how to organize the EHR data efficiently to match the structured inputs of a pretrained model such as BERT, and what are the appropriate domain-specific tasks for pretraining.

**Fig. 3:**
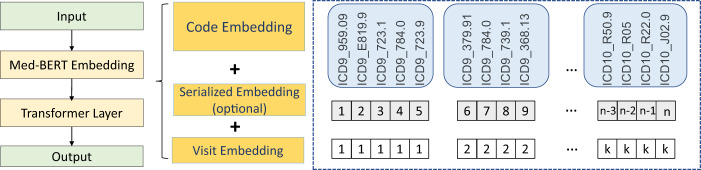
Med-BERT structure. The left part is a scheme of the overall Med-BERT architecture, the middle part details the individual components of the Med-BERT embedding layer, and the right part is an example of EHR data used in each embedding layer.

Figure 3 introduced our design of the Med-BERT embedding layers to accommodate the new modality. Specifically, three types of embeddings were taken as inputs for Med-BERT. These embeddings were projected from diagnosis codes, the order of codes within each visit, and the position of each visit and named, respectively, code embeddings, serialization embeddings, and visit embeddings. Code embeddings are the low-dimensional representations of each diagnosis code; serialization embeddings denote the relative order, in our case, the priority order, of each code in each visit; and visit embeddings are used to distinguish each visit in the sequence.

Unlike BERT, we did not use the specific tokens [CLS] and [SEP] at the input layer. Our choice is mainly due to the differences in the input formats of EHR and text. In BERT, only two adjacent sentences are fed for each input sample, and the token [SEP] serves as a separator of the two sentences for the pretraining task of next sentence prediction. Next sentence prediction, however, was not involved in our tasks (as explained in the next subsection). We reasoned that the visit embeddings can separate well each visit and that adding [SEP] would only be redundant. In BERT, the token [CLS] was used mainly to summarize the information from the two sentences. However, EHR sequences are usually much longer; e.g., a sequence may contain ten or more visits, and simply using one summarization token will inevitably lead to information loss. Therefore, for the classification tasks, either our prolonged LOS pretraining task or the downstream disease prediction tasks, where the information of a long-range sequence is usually needed, we added a feed-forward layer (FFL) to the sum of the output from all of the codes within visits to represent a sequence, instead of using only a single token. Of course, it is also possible to use an RNN prediction layer instead of a simple FFL on top of Med-BERT.

### Pretraining Med-BERT

We utilized the same optimization algorithm and recommended hyperparameters (see “Implementation details”) of the original BERT model^29^ during our Med-BERT pretraining phase. We trained the parameters of the Med-BERT model parameters on the diagnosis information of a cohort of 20 million patients using the following tasks.

#### Masked language model (Masked LM)

This task was directly inherited from the original BERT paper, which was used to predict the existence of any code, given its context. In detail, there was an 80% chance that a code was replaced by [MASK], a 10% chance that the code was replaced by a random code, and another 10% chance that it was kept unchanged. This task is the core of the contextualized embedding model.

#### Prediction of prolonged length of stay (Prolonged LOS) in hospital

For the classification task, instead of using the question–answer pairs as in BERT, we decided to choose a clinical problem with a relatively high prevalence in our pretraining dataset and one that is not disease-specific to ensure better generalizability of our pretrained model. The three most commonly used quality-of-care indicators, mortality, early readmission, and Prolonged LOS in hospital were selected and tested. Through comparison, we found that the mortality and the early readmission tasks are relatively easy: the model quickly converges to >99% accuracy. Therefore, we chose prolonged LOS, the task of assessing each patient for whether an incident of prolonged hospital visit (LOS > 7 days) had ever occurred throughout the entire EHR sequence of the patient, as a pretraining task. We used this simplified version of prolonged LOS prediction by targeting at the patient level rather than the visit level to reduce the pretraining complexity. Also, similar to the Masked LM task, we are not aiming to define a real future predicting task during the pretraining phase.

We found that the prolonged LOS task for pretraining leverages the bidirectional structure of Med-BERT. A prolonged LOS not only reflects the patient’s health status recorded in the past visits but also has an impact on the subsequent visits. On the other hand, tasks such as disease onset prediction or mortality always will be terminated at the last visit of the patient sequence, the input data of which can be constructed in only one direction.

### Applying Med-BERT for downstream prediction tasks by fine-tuning

Med-BERT, similar to BERT, follows the pretraining fine-tuning paradigm. The pretrained model itself only generates contextualized embedding for each input token. The model outputs a general purpose embedding and does not directly output any prediction labels. For any specific downstream prediction task, a classification layer (prediction head) needs to be added on top of the Med-BERT model. One can use a simple prediction head such as FFL on top of the sequential output from the final Med-BERT layer. For EHR predictive models, a commonly used prediction head is the RNN rolling over the output of token embeddings.

During fine-tuning, following the original BERT, we attached a prediction head on top of the Med-BERT architecture. The parameters of the Med-BERT part were loaded and initialized from the pretrained model, and then the parameters of both the Med-BERT part and the prediction head were updated by gradient descent. The input of the model was data from a disease-specific training cohort, which we referred to as the fine-tuning cohort. To understand the added values by the pretrained Med-BERT (especially the usefulness of big training data), we compared the results of fine-tuning the pretrained model and the untrained model (same architecture with a randomly initialized token + segment + position embedding layers and the multi-head transformer layers). All models were fine-tuned on a validation set (part of the fine-tuning cohort) and the reported numbers are the results on the test set.

### Evaluation of Med-BERT

We conducted evaluations on two disease prediction tasks on three cohorts from two databases. The two tasks are DHF and PaCa. We used Cerner for both tasks, forming the DHF-Cerner and PaCa-Cerner cohort; and used Truven for only the pancreatic cancer prediction task, forming the PaCa-Truven cohort, for generalizability evaluation. The detailed cohort definitions are presented in the “Methods” section. Unlike BEHRT and G-BERT, whose evaluation tasks are simply the prediction of certain codes which are similar to the tasks in pretraining, our definition of disease prediction tasks is more complex, as it requires the phenotyping from multiple perspectives, e.g., the existence of certain diagnosis codes, drug prescriptions, procedures, laboratory test results, and, sometimes, the frequency of events in predefined time windows. Therefore, we claim that our evaluation tasks are more realistic (compared with BEHRT) and more helpful in establishing the generalizability of Med-BERT.

For all three tasks, we conducted three experiments: (1) Ex-1: to evaluate how Med-BERT can contribute to state-of-the-art methods; (2) Ex-2: to compare Med-BERT with one state-of-the-art static clinical word2vec-style embedding, t-W2V (trained on the full Cerner cohort)^45^; and (3) Ex-3: to investigate how much the pretrained model can help in transfer learning with various training sample sizes.

For each fine-tuning task, we randomly selected a subset of the original cohort and further split it into training, validation, and testing sets with the ratio of 7:1:2. Since we have enough patients that are not included in the pretraining, we prioritized the assignment of samples to the test set to ensure that our test sets did not include any patient previously included in the Med-BERT pretraining set. For performance measurement, we used the AUC as our primary evaluation metric, which has been widely adopted by many previous studies of disease prediction^12,14,52^. Additional performance evaluation metrics are reported in Supplementary Tables 1 and 2.

For Ex-1, to evaluate the augmented power of pretrained Med-BERT on top of state-of-the-art base models, we compare the performances of the base models only and the performance of the base models on top of Med-BERT. We use GRU^53^, Bi-GRU^54^, and RETAIN^12^ as our base recurrent neural networks (RNN) models. While GRUs were shown to be very competitive baseline models, we also included RETAIN, a popular disease prediction model with double GRUs with attention. We also presented the results by using Med-BERT only; i.e., only FFL was added on top of the last layer of Med-BERT. This Med-BERT only model will provide an evaluation beyond RNN-based models. In addition, to evaluate the effect of pretraining using big data, we compare the performance of pretrained Med-BERT with the untrained Med-BERT architecture. For the sake of completeness, we also included L2-regularized logistic regression (L2LR) and random forest (RF), two popular non-deep learning methods, using standard multi-hot input format, as baseline models.

For Ex-2, to compare Med-BERT against static embeddings, we chose the t-W2V model. Our decision to use t-W2V to represent non-contextualized static embeddings was based on a previous study^45^ where different static embedding techniques including word2vec^24^, fasttext^55^, and pointwise positive mutual information-singular value decomposition^56^ were compared and t-W2V was found to perform best in the evaluated disease prediction task. Notably, Glove^25^ is a competent alternative of word2vec (w2c) for static EHR concept embedding but it was documented as having a comparable performance with w2c. Therefore, we selected t-W2V as our baseline for static embedding for the sake of convenience.

For Ex-3, to evaluate the value-added of Med-BERT with various fine-tuning training sizes, we selected samples with increasing sizes from the training data for each cohort for fine-tuning. Intuitively, the pretrained model would be more helpful when the training size is smaller, as it helps inject a broader scope of knowledge.

For Ex-1 and Ex-2, where we used the full fine-tuning training cohorts, we reported the average AUC and standard deviation for each model, based on ten runs with randomly initialized prediction head weights. For all iterations in Ex-3, we conducted a random bootstrap sampling ten times and reported the average AUC and standard deviation for each cohort.

### Performance boost of Med-BERT on fine-tuning tasks

**Fig. 4:**
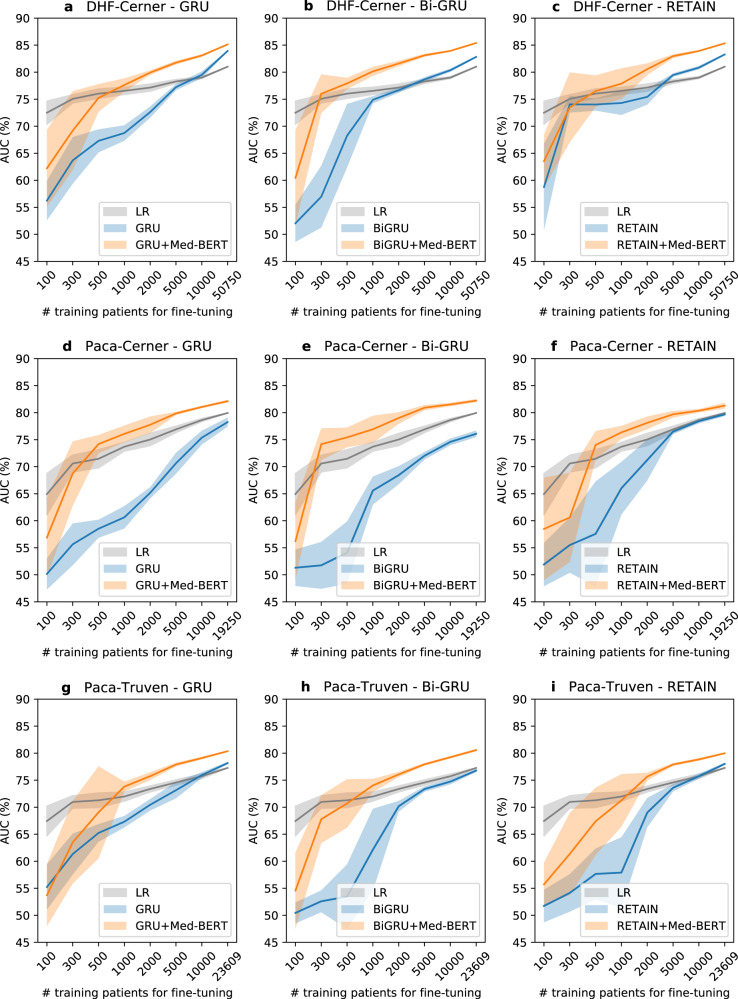
Comparison of prediction AUC for the test sets by training on different sizes of data on various cohorts between the methods with or without the pretrained Med-BERT layer. Logistic regression (LR) results are included as a baseline. **a** Cohort: DHF-Cerner, method: GRU; **b** cohort: DHF-Cerner, method: bidirectional GRU; **c** cohort: DHF-Cerner, method: RETAIN; **d** cohort: PaCa-Cerner, method: GRU; **e** cohort: PaCa-Cerner, method: bidirectional GRU; **f** cohort: PaCa-Cerner, method: RETAIN; **g** cohort: PaCa-Truven, method: GRU; **h** cohort: PaCa-Truven, method: bidirectional GRU; **i** cohort: PaCa-Truven, method: RETAIN. The shadows indicate the standard deviations.

Table 4 presents the AUCs for Ex-1 on the three fine-tuning evaluation tasks. The trends of additional performance evaluation metrics (Supplementary Tables 1 and 2) are largely consistent with that of AUC shown in Table 4 and Fig. 4. For DHF-Cerner, it is notable that Bi-GRU + Med-BERT and RETAIN + Med-BERT obtain the best results and perform comparably, followed by Med-BERT_only and GRU + Med-BERT. For each base model, adding t-W2V (except GRU) will generally achieve better results, but adding Med-BERT improves the results much further. It is remarkable that those powerful deep learning-based models, such as GRU, Bi-GRU, and RETAIN that already obtain over 0.83 on AUC with relatively large training data, e.g., 50 K samples, adding Med-BERT still makes a considerable performance boost.

**Table 4:** Average AUC values and standard deviations (in parentheses) for the different methods for the three evaluation tasks.

Model	DHF-Cerner	PaCa-Cerner	PaCa-Truven
GRU	83.93 (0.13)	78.26 (0.84)	78.17 (0.21)
GRU + t-W2V	83.95 (0.24)	80.08 (1)	77.54 (0.27)
GRU + Med-BERT	85.14 (0.06)	82.13 (0.24)	80.37 (0.12)
Bi-GRU	82.82 (0.17)	76.09 (0.61)	76.79 (0.29)
Bi-GRU + t-W2V	84.23 (0.06)	79.35 (0.27)	77.44 (0.22)
Bi-GRU + Med-BERT	**85.39** (**0.05)**	**82.23** (**0.29)**	**80.57** (**0.21)**
RETAIN	83.28 (0.16)	79.68 (0.32)	78.02 (0.19)
RETAIN + t-W2V	84.98 (0.02)	81.8 (0.17)	79.46 (0.18)
RETAIN + Med-BERT	85.33 (0.09)	81.3 (0.55)	79.98 (0.17)
Med-BERT_only (FFL)	85.18 (0.12)	81.67 (0.31)	79.98 (0.26)
untrained Med-BERT only	82.76 (0.13)	75.16 (0.77)	75.9 (0.18)
Logistic Regression (LR)^a^	81.01 (0)	79.94 (0)	77.28 (0)
Random Forest (RF)^a^	81.88 (0.08)	79.48 (0.31)	77.00 (0.12)

^a^LR and RF input is one hot representation while other models using embeddings.

The numbers in boldface indicate the highest AUROC per task.

For PaCa-Cerner, similar trends also were observed, whereby Bi-GRU + Med-BERT, Med-BERT_only, and GRU + Med-BERT generally outperform methods without Med-BERT and adding Med-BERT enhanced the AUCs of the base models by 1.62–6.14%. For PaCa-Truven, the best AUC was obtained by GRU + Med-BERT, whereas the other Med-BERT-related models also have better results than those without Med-BERT. On this Truven dataset, we still observe performance gains of 1.96–3.78%, although the average improved AUCs appear to be a bit lower than those on PaCa-Cerner. Nevertheless, it is reassuring to see that Med-BERT can be generalized well to a different dataset whose data distributions might be quite different from Cerner, the one it was pretrained on.

As an ablation experiment, we also made a comparison between the result of pretrained Med-BERT and that of untrained Med-BERT, where “untrained” means we did not feed the model with large EHR for a self-supervised pretraining but only took advantage of its structure. Table 4 shows that untrained Med-BERT performs much worse than Med-BERT only and does not even outperform the baseline method of logistic regression (LR) for PaCa prediction tasks. Therefore, we can conclude that the pretraining phase plays a more important role for the boosted performance. Cases where untrained Med-BERT does not outperform the baseline LR are likely due to overfitting, although we used the standard practice of both early stopping and dropout to reduce the likelihood of overfitting during the model training. This is possibly due to the fact that the untrained Med-BERT is an over-parameterized model (around 17 million parameters) with a huge number of configurations, so it might overfit to the training data^57^. On the other hand, the pretrained model started with a good configuration that is robust to a very large dataset for the pretraining, and thus is likely to generalize well.

It is a standard practice that the pretrained BERT model is not used on its own for prediction, rather a prediction head is needed for the fine-tuning tasks^29^. Since Med-BERT is an unsupervised pretraining model, fine-tuning should be done with certain configurations for different tasks, especially on the input data formats. However, in Table 4, we observed that a Med-BERT model with only an FFL on top of the last layer (Med-BERT_only (FFL)) can also obtain competitive performances.

In Fig. 4 we show how much Med-BERT can help boost the prediction performance of the base deep learning models by incorporating contextual information through pretraining. In the line chart of DHF-Cerner, we notice that, without Med-BERT, it is difficult for GRU only to have an AUC exceeding 0.65 when given fewer than 1000 training samples. The addition of Med-BERT, however, greatly increases the AUCs by about 20% and helps the model to reach 0.75, even when training on 500 samples. For Bi-GRU, considerable improvements also can be observed, but they are not as high as those for GRU. For RETAIN, Med-BERT seems to be more helpful when the training set contains more than 500 samples.

For PaCa-Cerner, large improvements by adding Med-BERT to GRU and Bi-GRU were demonstrated for almost all training sizes. In particular, for Bi-GRU, Med-BERT enables the AUC to reach 0.75 when training on only 300 samples. The charts for PaCa-Truven show similar trends, but the overall AUC values are lower compared to those on PaCa-Cerner when training on smaller sample sizes.

LR, a popular non-DL machine learning algorithm, serves consistently as a competitive baseline model, especially on small datasets. Indeed, for smaller training sizes as 500 or less in our experiment, L2LR showed decent performances. However, Med-BERT outperforms L2LR in all prediction tasks when the sample size is over 1000.

### Visualization of attention patterns in Med-BERT

Med-BERT not only offers improvement for prediction accuracy but also enables prediction interpretation. It is interesting and meaningful to explore how the pretrained model has learned using the complex structure and a huge volume of data. We show several examples of how codes are connected with each other according to the attention weights from the transformer layers, the core component of Med-BERT.

The bertviz tool^58^ was adapted and improved to better visualize the attention patterns in each layer of the pretrained model. We added “SEP” tokens between visits only for visualization purposes. We observed distinct patterns in different layers of the model. In the pretrained model, among the six layers of the BERT transformer model, the connections of the first two layers are mostly syntactic, some attention heads are restricted within a visit, and some point to the same codes across different visits. In the middle two layers, some medically meaningful attention patterns that capture contextual and visit-dependent information emerge. For the final couple of layers, the attention patterns become diffused and difficult to interpret.

**Fig. 5:**
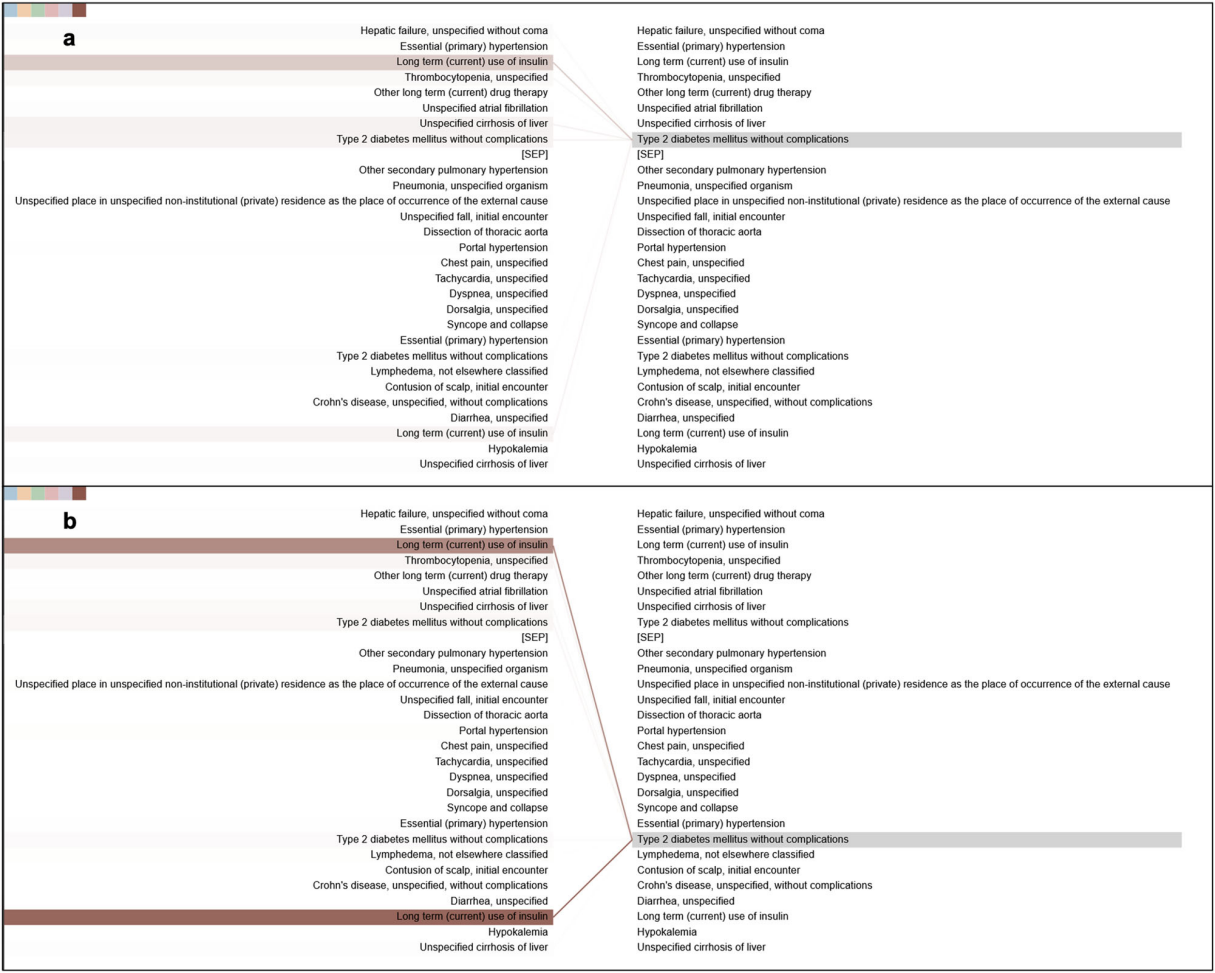
Example of different connections of the same code, “type 2 diabetes mellitus”, in different visits. **a** The first visit, **b** the second visit. Connection lines from the code in the left panel to the code in the right panel indicate attentions of the Med-BERT model. Color of the line indicates individual attention head, and intensity of the line indicates attention weights.

Figure 5 is an example of the same code in different visits, showing different attention patterns. This demonstrates the ability of Med-BERT to learn contextualized representations. The earlier code for type 2 DM focuses mainly on the code for the long-term use of insulin within the same visit, but the later diabetes code focuses on the insulin code, both in the current and the previous visits. This could potentially indicate that the model learns the temporal relationship between visits through the segment embedding. More examples are provided in Supplementary Fig. 3.

**Fig. 6:**
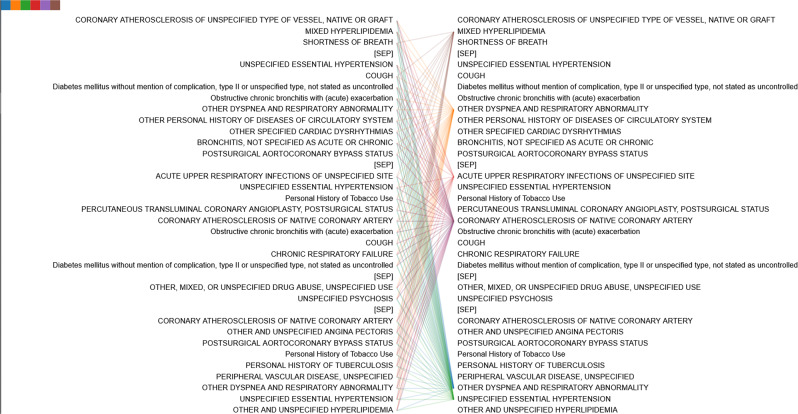
Example of the dependency connections in the DHF-Cerner cohort. Connection lines from the code in the left panel to the code in the right panel indicate attentions of the Med-BERT model. Color of the line indicates individual attention head, and intensity of the line indicates attention weights.

**Fig. 7:**
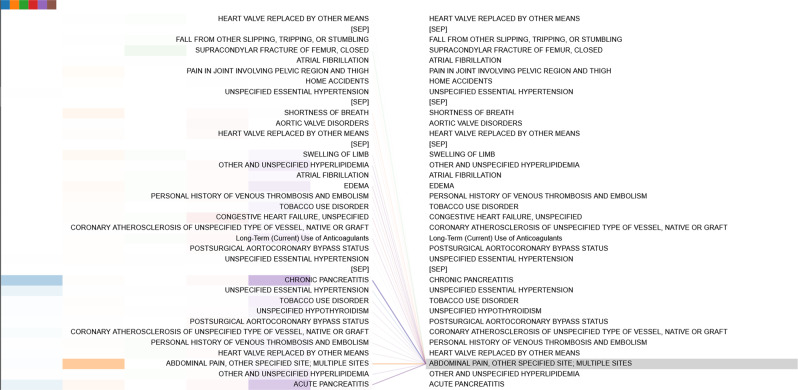
Example of the dependency connections in the PaCa-Cerner cohort. Connection lines from the code in the left panel to the code in the right panel indicate attentions of the Med-BERT model. Color of the line indicates individual attention head, and intensity of the line indicates attention weights.

The attention patterns of the fine-tuned model are different. The fine-tuned models express distinct task-dependent patterns across different layers, showing the generalizability and adaptability of the model for learning different levels of knowledge in real-world scenarios. Figure 6 provides an example of the Med-BERT model fine-tuned on the DHF-Cerner dataset with attention converging onto several related codes in the second layer. Figure 7 is an example of the attention pattern in the fourth layer of the Med-BERT model fine-tuned on the PaCa-Cerner dataset, capturing the relevant correlation between diagnosis codes. Additional visualization patterns can be seen in Supplementary Fig. 3. We believe that these kinds of visualization patterns can help us to better understand the inner mechanism of the neural network model and to build trusting and better communications of health information.

## Discussion

Med-BERT shows its power in helping to improve the prediction performance on multiple tasks with different configurations, and it is particularly effective in the “extreme transfer learning” paradigms, i.e., fine-tuning on only several hundreds of samples. Deep learning-based predictive models usually require at least thousands of samples. These models need to learn complex semantics through feeding samples that convey different underlying disease progressions and variational context information so that they can be capable of dealing with intricate unseen cases. However, most deep learning algorithms are insufficient in modeling the data comprehensively due to their limitation in an in-depth understanding of the inputs. Pretrained models can well address this issue by using more sophisticated structures to better capture the complex semantics of inputs, behaving as a knowledge container, and injecting the knowledge into new tasks. Similar to pretrained models on other domains, Med-BERT, by using its bidirectional transformer and deep structure as well as big data, also have been shown in this study to be extremely helpful when transferring to new tasks.

Masked LM and Prolonged LOS were designed and included to reinforce the modeling of contextual information and to help collect sequential dependencies. Labels for both can be generated in an unsupervised way, i.e., without human annotations. In Masked LM, the goal is to predict a masked code using the sequential information from the forward and the backward directions. In Prolonged LOS, the goal is to determine whether a patient is associated with any visit that is a prolonged stay, which also relies on cumulative contexts. We believe that, by including the prediction tasks from both the code level and the patient (sequence) level, Med-BERT can further strengthen the representation learning of EHR sequences from different granularities.

Intuitively, a better parameter initialization of deep learning models could lead to better performance and faster convergence. However, these benefits would gradually diminish with the growth of training samples. We consider 50 and 20 K as acceptable scales of samples for training satisfactory (converging) deep learning models. When we added Med-BERT, however, considerable improvements also could be observed. For example, RETAIN obtains satisfactory performances on all the three tasks, but adding Med-BERT brings further improvements by 1.62–2.05%. In addition, for GRU and Bi-GRU, whose model structures are simpler than that of RETAIN, the improvements can be much larger, which bring these simple models to a comparable level of or even better than RETAIN. Further, according to the results of Med-BERT_only, which also achieves good performance, we may conclude that Med-BERT will potentially release researchers from developing complex models for disease prediction problems.

Similar to Med-BERT, static embedding method t-W2V also can serve as a good performance booster to the base deep learning models. However, the improvements of t-W2V are smaller compared to Med-BERT in most cases. A probable explanation is that t-W2V has limitations in modeling long-sequential information, considering its shallow structure and the limited size of the context window which cannot be guaranteed to act well in all situations.

In practice, Med-BERT will significantly help to reduce the burden of data labeling, which can be seen through comparing the sizes of training samples required to achieve certain AUC levels. Ex-3 proved the effectiveness of transferring Med-BERT into realistic disease prediction tasks. Most of the charts in Fig. 4 reflect that Med-BERT can substantially boost the performance of base models on small samples. For example, in the first sub-chart of PaCa-Cerner in Fig. 4, if we draw a horizontal line across the y-tick of 0.75, we will see a requirement of 1000 samples for GRU + Med-BERT and over 10,000 samples for GRU only. Similarly, we can see the Bi-GRU + Med-BERT trained on 5000 samples can provide slightly better performance than Bi-GRU only trained on more than 50,000 samples as appears in Supplementary Table 2A.

Thus, Med-BERT brought the model performance on par with a training set almost ten times larger. The data acquisition cost of these over 9000 samples, which sometimes can be quite expensive, will be substantially saved by using Med-BERT. In this situation, with Med-BERT, researchers and clinicians are able to quickly get a general and acceptable understanding of the progressions of new diseases before collecting enough annotated samples.

Admittedly, although Med-BERT empowers deep learning models throughout all training sample sizes tested, Med-BERT powered models still do not outperform the non-deep learning baseline model LR for the smallest training sample sizes (*n* < 500). This is consistent with the literature that LR remains a competitive predictive model for small training sample sizes in a number of studies^14^. LR benefits from its simple and shallow structure, which is much easier to fit based on even only a few samples compared with the complex structure and immense parameter space of deep learning models. However, this advantage is gradually weakened as the training size grows. Therefore, for practice, we would recommend the use of Med-BERT fine-tuning for the scenarios where the training sample size is sufficiently large (e.g., *n* > 500).

The vocabulary of the current version of Med-BERT is the union of ICD-9 and ICD-10 codes with 82,000 tokens. Compared with BEHRT and G-BERT, our vocabulary has broader coverage and is widely adopted in practice. We believe that it will greatly facilitate the transferability of the model, as ICD is a global health information standard recommended by the World Health Organization and is used by different institutions from over 100 countries around the world. This can be demonstrated in our PaCa-Truven evaluation, in which we tested our models’ efficacy using a cohort extracted from a health insurance dataset.

In this work, we chose BERT, an advanced contextualized embedding methodology in NLP, for EHR modality. However, there are alternative ideas: such as ULMFiT^46^, ELMo^26^ GPTs^27,28,59^, etc. It is probably necessary to evaluate these alternatives for pretraining and fine-tuning on EHR. We will leave it as future work.

There are still several limitations of the current work. First, we used only the diagnosis information in the ICD format. Second, we did not include the length of time intervals between visits in this study, which may cause some temporal information loss. Third, we did not fully explore the order of concepts within each visit, and the current setting based on code priorities might not be sufficiently reliable. In the future, more research on designing different pretraining tasks will be conducted, and different types of fine-tuning tasks beyond disease prediction also will be tested. We also plan to include other sources, such as time, medications, procedures, and laboratory tests, as inputs of Med-BERT. In addition, task-specific visualizations and interpretations are other areas that we plan to explore.

In conclusion, we proposed Med-BERT, a contextualized embedding model pretrained on a large volume of structured EHR data, and further evaluated the model in disease prediction tasks. Domain-specific input formats and pretrained tasks were designed. Extensive experiments demonstrated that Med-BERT has the capacity to help boost the prediction performance of baseline deep learning models on different sizes of training samples and can obtain promising results. The visualization module enabled us to look deeper into the underlying semantics of the data and working mechanisms of the model, in which we observed meaningful examples. Those examples were further verified by clinical experts, indicating that Med-BERT can capture the semantics among EHRs during both pretraining and fine-tuning. Methodologically, our work establishes the feasibility and usefulness of contextualized embedding of structured EHR data. Practically, our pretrained model enables training powerful deep learning predictive models with limited training sets.

## Methods

### Med-BERT pretraining cohort

Cerner Health Facts^®^ (version 2017) is a de-identified EHR database that consists of over 600 hospitals and clinics in the United States. It represents over 68 million unique patients and includes longitudinal data from 2000 to 2017. The database consists of patient-level data, including demographics, encounter meta-information, diagnoses, procedures, lab results, medication orders, medication administration, vital signs, microbiology, surgical cases, other clinical observations, and health systems attributes. Data in Health Facts^®^ are extracted directly from the EMRs of hospitals with which Cerner has a data use agreement. Encounter meta-information includes the identification of pharmacy, clinical and microbiology laboratory, and admission and billing information from affiliated patient care locations. All admissions, medication orders and dispensing, laboratory orders, and specimens are date and time-stamped, providing a temporal relationship between treatment patterns and clinical information. The Cerner Corporation has established Health Insurance Portability and Accountability Act-compliant operating policies to establish de-identification for Health Facts^®^.

During the data preprocessing phase for pretraining, for each patient in the cohort, we organized the visits in a temporal order and ranked the diagnosis codes within each visit according to three criteria: (1) the diagnosis was flagged as present on admission; (2) the diagnosis was captured during the visit (e.g., hospitalization) or only at the billing phase; and (3) the diagnosis priority is provided by the Cerner database, indicating some priorities of the diagnoses, e.g., principal/secondary diagnosis (the priority is provided by the database, but it might not be a perfect priority ranking)

For each visit, we extracted the diagnosis codes (represented by ICD, Ninth Revision, Clinical Modification (ICD-9) and ICD, Tenth Revision, Clinical Modification (ICD-10)) and the length of stay in hospital. We then ranked the codes in each visit according to the above three criteria and determined the order by using (1) → (2) → (3) in sequence. We observed only very limited performance gains, however, by adding the code order during the evaluation, compared with randomly scattering the codes. Hence, we set it as a placeholder here and assume that more effective orders could be defined in the future.

Patients with fewer than three diagnosis codes in their records as well as those with wrong recorded time information, e.g., discharge date before admission date, were removed from the population. In total, we had 28,490,650 unique patients (Fig. 1), which were further separated into training, valid, and testing sets by the ratio of 7:1:2 on both the pretraining and evaluation phases.

### Diabetes heart failure cohort (DHF)

We originally identified 3,668,780 patients with at least one encounter with a diabetes diagnosis, based on the associated ICD-9/10 codes. We decided to exclude patients with any history of diabetes insipidus, gestational diabetes, secondary diabetes, neonatal diabetes mellitus (DM), or type I DM from our cohort, as we focus on patients with type II DM and need to avoid any chance of wrong coding, taking into consideration that most of the EHR data are based on user manual entries and that there is a high associated chance of data entry mistakes. For the same reason, we decided to include patients who have more than one encounter with a diabetes diagnosis code. In addition, for type II DM patients, we verified that the patients’ A1C reading is ≥6.5 or that they are taking an antidiabetic agent, including metformin, chlorpropamide, glimepiride, glyburide, glipizide, tolbutamide, tolazamide, pioglitazone, rosiglitazone, sitagliptin, saxagliptin, alogliptin, linagliptin, repaglinide, nateglinide, miglitol, acarbose, or insulin.

For these cases, we identified patients with incidences of heart failure (HF) (using ICD-9 code equivalents, such as 428, or in 404.03, 404.13, 402.11, 404.11, 402.01, 404.01, 402.91, 398.91, 404.93, and 404.91, or ICD-10 code equivalents, such as I50%, or in I11.0, I09.81, I13.2, I97.13, I97.131, I13.0, and I97.130). In addition, we verified that the eligible cases are either prescribed a diuretic agent, had high B-type natriuretic peptide or had been subjected to relevant procedures, including dialysis or an artificial heart-associated procedure following^60^. We included only those patients who reported HF at least 30 days after their first encounter with a type II DM code and excluded patients with only one HF encounter.

Further data cleaning included the exclusion of patients with incorrect or incomplete data, for example, patients who were recorded as expired in between their first encounter and our event (first HF encounter for cases or last encounter for controls) as well as patients who are younger than 18 years old at their first diabetes diagnosis. The final cohort is shown in Supplementary Fig. 1 and includes 39,727 cases and 632,920 controls.

### Pancreatic cancer cohort (PaCa)

Using ICD-9 codes that start with 157 and ICD-10 codes that start with C25, we originally identified around 45,000 pancreatic cancer patients from the Cerner Health Facts dataset, of which 11,486 cases of individuals of 45 years or older did not report any other cancer disease before their first pancreatic cancer diagnosis were eligible for inclusion in this cohort. Further details of the cohort definition are shown in Supplementary Fig. 2.

Similarly, we extracted a PaCa cohort from Truven Health MarketScan^®^ Research Databases for evaluation purposes. The Truven Health MarketScan^®^ Research Databases (version 2015) are a family of research datasets that fully integrate de-identified patient-level health data (medical, drug, and dental), productivity (workplace absence, short- and long-term disability, and workers’ compensation), laboratory results, health risk assessments, hospital discharges, and electronic medical records into datasets available for healthcare research. It captures person-specific clinical utilization, expenditures, and enrollment across inpatient, outpatient, prescription drug, and carve-out services. The annual medical databases include private sector health data from ~350 payers. Historically, more than 20 billion service records are available in the MarketScan databases. These data represent the medical experience of insured employees and their dependents for active employees, early retirees, Consolidated Omnibus Budget Reconciliation Act continues, and Medicare-eligible retirees with employer-provided Medicare Supplementary plans. Most of the diagnosis codes in Truven are ICD-9 codes, as the version of the database that we used is 2015, but the implementation of ICD-10 started in October 2015^61^.

### Implementation details

For the transformer architecture of Med-BERT, we used six layers, six attention heads, and a hidden dimension of 192 (*L* = 6, *H* = 192, *A* = 6). We set the feed-forward/filter size to be 64.

For pretraining, we set the maximum sequence length as 512 tokens. We masked one diagnosis code per patient during Masked LM. We used the default BERT optimizer, AdamWeight decay optimizer. We used the recommended learning rate of 5e−5, and a dropout rate of 0.1. We used the TensorFlow code of the original BERT from https://github.com/google-research/bert (February 2019 version). We used a single Nvidia Tesla V100 GPU of 32 GB graphics memory capacity, and we trained the model for a week for more than 45 million steps, for which each step consists of 32 patients (batch size).

Before fine-tuning, we first converted the pretrained model to the PyTorch version, using the HuggingFace package (version 2.3)^62^. For fine-tuning, we utilized our established codebase https://github.com/ZhiGroup/pytorch_ehr for the implementation of BERT_only, GRU, bi-GRU, and RETAIN models with minor modification to implement multilayer embeddings instead of visit-level embeddings. We used the Adam optimizer and a learning rate of 1e−5 for most of the models except for unidirectional GRU with static embedding for which a learning rate of 0.001 was associated with the best results. For the evaluation tasks, we used Nvidia GeForce RTX 2080 Ti GPUs of 12 GB memory.

For L2LR and RF, we used the scikit-learn package version 0.24. We used the default hyperparameters for both the LR and the RF classifiers.

### On ethical data use related to this manuscript

The IBM^®^ MarketScan^®^ Research Databases (Formerly, Truven^®^) contain individual-level, de-identified, healthcare claims information from employers, health plans, hospitals, and Medicare and Medicaid programs. The data in Health Facts^®^ are extracted directly from the EMR of hospitals with which Cerner has a data use agreement. Both IBM and Cerner Corporation have established Health Insurance Portability and Accountability Act-compliant operating policies to establish de-identification for IBM^®^ MarketScan^®^ Research Databases and Health Facts^®^. The use of IBM^®^MarketScan^®^ Research Databases and Cerner Health Facts^®^ mandates compliance with all vendor contractual obligations; of specific ethical relevance is the legally binding directive that no user of these data may attempt to re-identify the de-identified data. As an additional safeguard, at an institutional level, UTHealth researchers employing the IBM^®^ MarketScan^®^ Research Databases and Cerner Health Facts^®^ for their studies are subject to oversight and approval by the Committee for the Protection of Human Subjects (UTHSC-H IRB) under protocol HSC-SBMI-13-0549. The use of the IBM^®^ MarketScan^®^ Research Databases and Cerner Health Facts^®^ for this study is covered by the approval by the Committee for the Protection of Human Subjects (UTHSC-H IRB) under protocol HSC-SBMI-13-0549.

### Reporting summary

Further information on research design is available in the Nature Research Reporting Summary linked to this article.

## Supplementary information

Supplementary Information

Reporting Summary

## Data Availability

The data that support the findings of this study are available from the Data Service Office at the University of Texas Health Science Center at Houston School of Biomedical Informatics (SBMI) but restrictions apply to the availability of these data, which were used under license from the data provider.

## Code availability

To facilitate reproducibility and benefit other EHR-based studies, we shared our source code as well as our visualization tool on https://github.com/ZhiGroup/Med-BERT. The pretrained models are available from the authors upon request and with permission of the SBMI Data Service Office.
