# Supplementary material for: Med-BERT: pretrained contextualized embeddings on large-scale structured electronic health records for disease prediction
Source: NPJ Digit Med. 2021 May 20;4:86. doi: 10.1038/s41746-021-00455-y (PMC8137882; doi:10.1038/s41746-021-00455-y)
Supplement: Supplementary file 1 — Supplementary Information [file 41746_2021_455_MOESM1_ESM.pdf]

Supplementary information for
Med-BERT: pre-trained contextualized embeddings on large-scale structured electronic health records for disease prediction

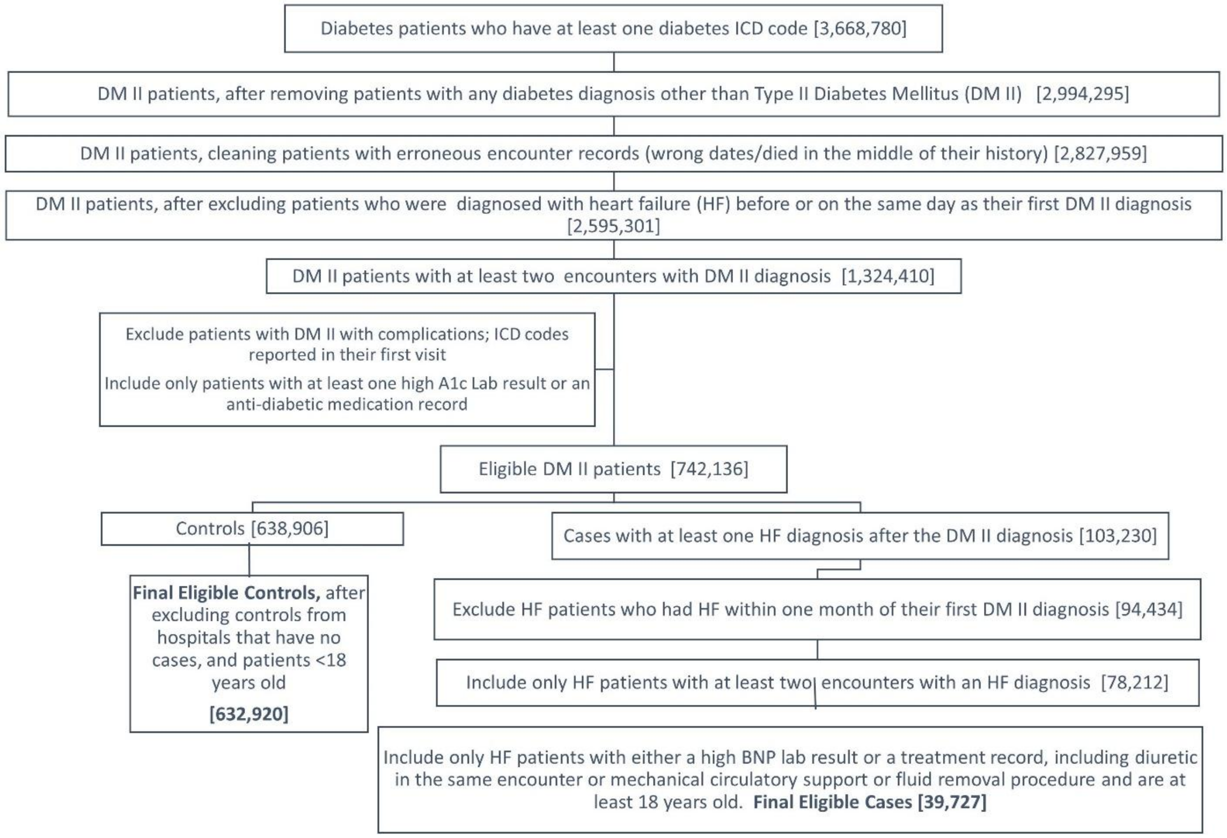

Supplementary Figure 1. Flowchart for the DHF cohort definition.

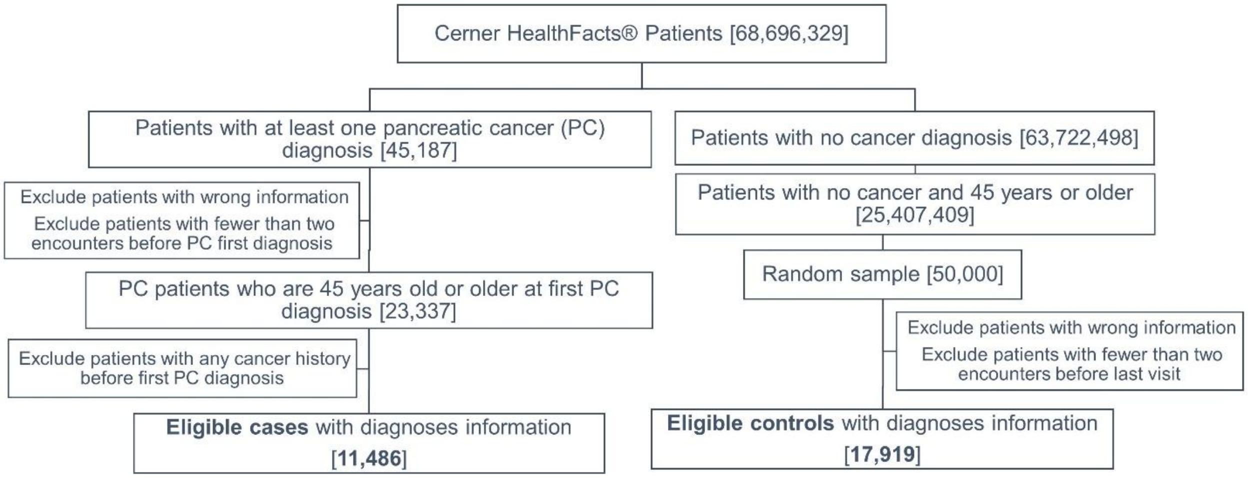

Supplementary Figure 2. Flowchart for the PaCa cohort definition.

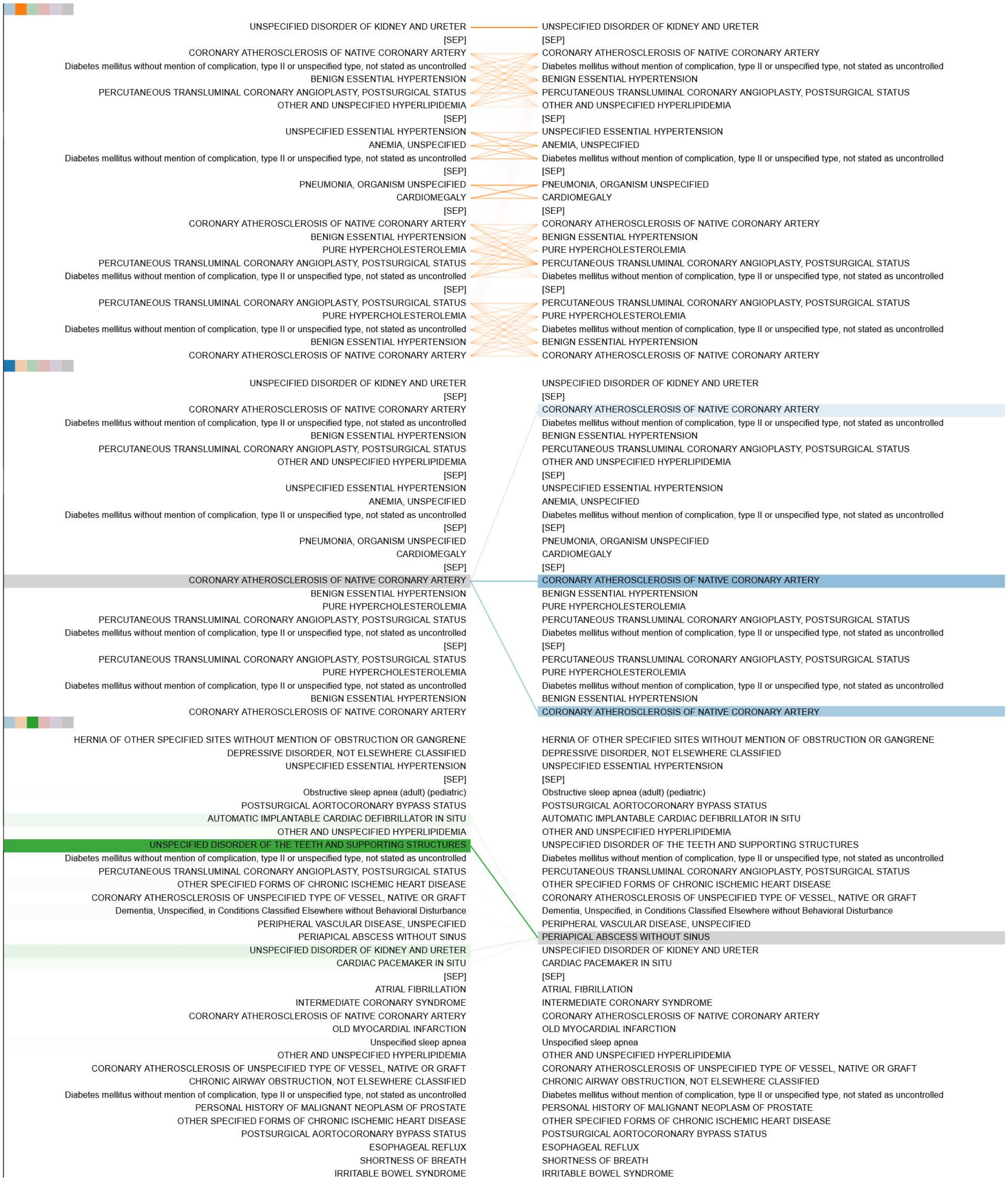

Supplementary Figure 3. Attention connections from the first three transformer layers (a top-down direction) of a sample patient sequence. In the first layer, several heads show short-range attention patterns, and each token attends mainly to the nearby tokens that are within the same visit. In the second layer, some attention heads learn to make the correspondence between the same tokens. The third layer has the most interpretable patterns. A token in the third layer will focus strongly on other relevant tokens but mostly within the same visit. After the third layer, the attention becomes more diffuse and less explainable; however, there are still some heads that show long-range attention patterns.

Supplementary Table 1: Additional benchmark results.

Supplementary Table 1A: Average values and standard deviations (in parentheses) of additional evaluation metrics for DHF prediction

Model	Validation AUC	AUPRC	Sensitivity	Specificity	Precision (PPV)	F1-score
GRU	84.22 (0.09)	82.07 (0.19)	78.26 (4.37)	73.28 (4.35)	74.53 (2.16)	76.22 (1.1)
GRU+t-W2V	84.25 (0.15)	82.23 (0.26)	78.18 (3.41)	73.39 (3.28)	74.51 (1.5)	76.23 (0.85)
GRU+Med-BERT	85.29 (0.12)	83.44 (0.13)	81.25 (1.64)	72.37 (2.09)	74.47 (1.06)	77.69 (0.25)
Bi-GRU	83.04 (0.18)	81.32 (0.18)	77.07 (1.15)	72.72 (1.14)	73.68 (0.56)	75.33 (0.34)
Bi-GRU+t-W2V	84.59 (0.12)	82.66 (0.1)	80.60 (1.50)	71.70 (1.53)	73.85 (0.7)	77.06 (0.34)
Bi-GRU+Med-BERT	85.39 (0.07)	83.87 (0.05)	79.51 (2.50)	75.21 (2.53)	76.12 (1.3)	77.74 (0.56)
RETAIN	83.44 (0.25)	81.35 (0.16)	77.20 (0.62)	73.70 (0.63)	74.41 (0.33)	75.77 (0.21)
RETAIN+t-W2V	85.17 (0.06)	83.34 (0.05)	79.79 (0.78)	73.76 (0.85)	75.08 (0.43)	77.36 (0.16)
RETAIN+Med-BERT	85.36 (0.11)	83.63 (0.11)	78.07 (2.73)	76.09 (2.49)	76.44 (1.26)	77.2 (0.74)
Med-BERT_only (FFL)	85.25 (0.14)	83.67 (0.18)	78.09 (3.83)	75.87 (3.82)	76.35 (2.01)	77.12 (0.88)
untrained Med-BERT only	83.10 (0.22)	81.15 (0.17)	76.67 (2.73)	72.94 (2.37)	73.78 (1.03)	75.15 (0.81)
*Logistic Regression (LR)**	81.22 (0)	78.52 (0)	77.12 (0)	70.83 (0)	72.36 (0)	74.66 (0)
*Random Forest (RF)**	81.91 (0.35)	79.89 (0.17)	77.51 (0.11)	70.94 (0.43)	72.54 (0.28)	74.94 (0.15)

Supplementary Table 1B: Average values and standard deviations (in parentheses) of additional evaluation metrics for PaCa prediction using Cerner cohort

Model	Validation AUC	AUPRC	Sensitivity	Specificity	Precision (PPV)	F1-score
GRU	81.63 (0.30)	71.40 (0.90)	58.42 (2.53)	83.54 (2.61)	62.76 (2.99)	60.41 (0.98)
GRU+t-W2V	83.41 (0.13)	72.18 (1.22)	58.27 (4.40)	84.41 (5.14)	64.53 (5.43)	60.92 (1.63)
GRU+Med-BERT	84.06 (0.21)	74.84 (0.19)	64.52 (1.71)	82.86 (1.63)	63.99 (1.61)	64.22 (0.4)
Bi-GRU	79.65 (0.56)	69.05 (0.56)	57.09 (1.67)	82.36 (1.27)	60.41 (1.22)	58.67 (0.74)
Bi-GRU+t-W2V	82.75 (0.12)	71.98 (0.35)	59.49 (1.36)	84.53 (1.02)	64.45 (1.08)	61.85 (0.44)
Bi-GRU+Med-BERT	84.32 (0.13)	75.08 (0.36)	63.82 (2.75)	83.59 (2.81)	64.89 (2.86)	64.25 (0.69)
RETAIN	80.99 (0.32)	72.02 (0.30)	52.78 (1.76)	88.35 (0.79)	68.1 (1.03)	59.45 (1.04)
RETAIN+t-W2V	84.60 (0.18)	74.88 (0.20)	61.98 (1.62)	85.86 (1.43)	67.42 (1.71)	64.55 (0.61)
RETAIN+Med-BERT	83.34 (0.13)	71.78 (3.10)	59.24 (9.26)	84.94 (6.43)	66.36 (6.51)	61.71 (2.43)
Med-BERT_only (FFL)	83.96 (0.23)	73.91 (0.53)	65.03 (4.80)	80.53 (5.27)	61.79 (5.04)	63.03 (0.43)
untrained Med-BERT only	79.56 (0.57)	67.97 (0.91)	54.81 (4.19)	82.01 (4.61)	59.47 (4.61)	56.78 (1.38)
Logistic Regression (LR)	79.45 (0)	73.59 (0)	56.82 (0)	89.49 (0)	71.79 (0)	63.43 (0)
Random Forest (RF)	79.05 (0.08)	65.65 (0.33)	63.83 (0.27)	79.96 (0.53)	59.99 (0.66)	61.85 (0.40)

Supplementary Table 1C: Average values and standard deviations (in parentheses) of additional evaluation metrics for PaCa prediction using Truven cohort

Model	Validation AUC	AUPRC	Sensitivity	Specificity	Precision (PPV)	F1-score
GRU	77.31 (0.35)	68.93 (0.19)	49.54 (4.02)	88.06 (2.67)	68.2 (2.92)	57.2 (1.61)
GRU+t-W2V	77.19 (0.28)	67.90 (0.33)	43.30 (4.78)	90.98 (2.16)	71.35 (2.82)	53.64 (2.99)
GRU+Med-BERT	79.33 (0.17)	71.21 (0.28)	56.81 (2.00)	86.07 (1.32)	67.56 (1.31)	61.68 (0.66)
Bi-GRU	76.66 (0.21)	66.87 (0.32)	46.65 (0.81)	88.61 (0.63)	67.61 (1.05)	55.2 (0.58)
Bi-GRU+t-W2V	77.21 (0.29)	67.35 (0.27)	46.63 (1.81)	89.38 (1.07)	69.17 (1.36)	55.67 (0.93)
Bi-GRU+Med-BERT	79.45 (0.22)	71.54 (0.45)	56.80 (1.50)	86.02 (0.96)	67.45 (0.99)	61.65 (0.54)
RETAIN	77.80 (0.20)	68.93 (0.35)	45.74 (0.63)	90.24 (0.54)	70.5 (0.96)	55.48 (0.4)
RETAIN+t-W2V	79.58 (0.29)	70.36 (0.34)	51.41 (0.90)	88.76 (0.48)	69.99 (0.61)	59.27 (0.49)
RETAIN+Med-BERT	79.20 (0.16)	69.39 (0.97)	33.43 (8.33)	95.49 (2.83)	80.56 (5.16)	46.4 (7.02)
Med-BERT_only (FFL)	79.26 (0.19)	71.16 (0.59)	50.68 (5.43)	88.78 (3.33)	70.33 (4.23)	58.55 (2.45)
untrained Med-BERT only	75.89 (0.49)	65.56 (0.52)	47.75 (5.50)	86.23 (3.68)	64.34 (3.16)	54.5 (2.26)
Logistic Regression (LR)	77.11 (0)	67.33 (0)	45.52 (0)	89.17 (0)	68.16 (0)	54.58 (0)
Random Forest (RF)	76.36 (0.07)	64.62 (0.23)	43.28 (0.34)	89.71 (0.17)	68.17 (0.52)	52.94 (0.41)

Supplementary Table 2: Additional performance results

A. Experiement 3 - Additional Metrics for DHF prediction evalution using smaller training set size

Threshold used for Sensitivity, Specificity, Precision and F1-score is 0.5

Model	Training Set size	TEST AUC	Validation AUC	AUPRC	Sensitivity	Specificity	Precision (PPV)	F1-score
GRU	100	56.24 (3.65)	65.52 (24.07)	54.78 (3.42)	58.08 (8.72)	51 (10.08)	54.18 (3.31)	55.74 (4.16)
	200	62.31 (3.87)	70.79 (12.39)	60.73 (3.36)	60.62 (6.09)	57.75 (7.07)	58.83 (3.06)	59.55 (3.48)
	300	63.67 (4.32)	69.79 (9.37)	61.67 (3.83)	61.94 (6.07)	58.26 (7.56)	59.7 (3.63)	60.62 (3.66)
	400	64.34 (4.26)	68.52 (5.67)	62.26 (4.25)	58.77 (4.52)	62.09 (6.13)	60.7 (3.41)	59.62 (3.19)
	500	67.29 (2.13)	70.88 (6.25)	65.27 (1.92)	61.1 (5.71)	64.22 (5.22)	62.95 (2.15)	61.85 (2.97)
	1000	68.74 (1.43)	70 (4.82)	66.53 (1.54)	64.18 (6)	62.82 (5.89)	63.26 (1.92)	63.52 (2.46)
	2000	72.62 (1.03)	74.1 (4.03)	70.27 (0.96)	67.89 (2.06)	65.49 (3.28)	66.14 (1.45)	66.97 (0.66)
	5000	77.24 (0.58)	78.23 (1.07)	74.48 (0.5)	71.5 (5.88)	69.69 (4.72)	70.17 (1.55)	70.66 (2.16)
	10000	79.51 (0.63)	80.11 (1.32)	76.92 (0.85)	73.05 (4.94)	72.17 (4.39)	72.37 (1.82)	72.57 (1.64)
	Full Cohort (50750)	83.93 (0.13)	84.22 (0.09)	82.07 (0.19)	78.26 (4.37)	73.28 (4.35)	74.53 (2.16)	76.22 (1.1)
GRU+Med-BERT	100	62.21 (7.12)	71.1 (14.17)	60 (6.99)	65.58 (39.99)	42.27 (38.69)	50.43 (19.05)	50.45 (27.67)
	200	68.19 (7.47)	78.01 (9.82)	65.62 (6.79)	28.21 (33.51)	86.43 (16.3)	57.96 (21.78)	29.7 (32.39)
	300	69.18 (7.3)	73.69 (10.49)	65.67 (7.29)	75.08 (30.49)	44.23 (38.81)	59.25 (9.88)	61.28 (21.39)
	400	72.76 (3.97)	78.67 (6.02)	69.72 (4.35)	47.73 (31.95)	77.73 (17.05)	69.57 (5.55)	48.87 (29.09)
	500	75.24 (2.56)	79.11 (4.91)	71.96 (2.29)	73.76 (10.13)	62.18 (17.56)	67.2 (6.19)	69.53 (3.22)
	1000	77.63 (1.22)	76.6 (3.87)	74.55 (1.13)	74.93 (2.82)	67.66 (3.19)	69.71 (1.62)	72.18 (1.21)
	2000	79.92 (0.42)	80.43 (2.74)	77.51 (0.55)	75.31 (2.95)	70.37 (3.41)	71.65 (1.61)	73.37 (0.69)
	5000	81.76 (0.34)	81.92 (1.42)	79.36 (0.52)	78.61 (2.49)	69.76 (2.45)	72.07 (1.04)	75.16 (0.66)
	10000	83.08 (0.24)	83.5 (1.22)	81.01 (0.31)	79.32 (1.9)	71.16 (2.28)	73.18 (1.07)	76.1 (0.37)
	Full Cohort (50750)	85.14 (0.06)	85.29 (0.12)	83.44 (0.13)	81.25 (1.64)	72.37 (2.09)	74.47 (1.06)	77.69 (0.25)
Bi-GRU	100	52.04 (3.44)	54.83 (21.76)	51.73 (2.92)	54.34 (27.3)	48.41 (26.9)	51.24 (2.44)	49.02 (16.09)
	200	58.24 (3.43)	67.59 (7.24)	56.43 (2.67)	51.89 (16.31)	59.46 (12.33)	55.78 (1.97)	52.44 (10.56)
	300	56.96 (5.72)	61.76 (14.29)	55.36 (5.21)	59.42 (17.69)	50.13 (18.55)	54.54 (3.91)	55.35 (10.09)
	400	65.03 (4.94)	70.22 (10.63)	61.92 (4.49)	61.26 (6.88)	60.18 (7.57)	60.56 (3.68)	60.69 (4.08)
	500	68.24 (5.87)	70.51 (8.77)	65.39 (5.36)	61.76 (13.31)	64.48 (5.31)	62.86 (3.76)	61.82 (8.77)
	1000	74.89 (0.67)	72.33 (3.93)	72.7 (1.05)	70.2 (2.91)	66.32 (2.54)	67.4 (0.81)	68.73 (1.08)
	2000	76.66 (0.46)	78.54 (3.89)	74.75 (0.5)	70.79 (1.69)	69.02 (1.91)	69.38 (0.93)	70.06 (0.67)
	5000	78.66 (0.41)	79.71 (1.14)	77.03 (0.53)	73.06 (1.9)	69.58 (1.99)	70.43 (0.86)	71.7 (0.57)
	10000	80.36 (0.31)	80.72 (1.31)	78.68 (0.33)	74.58 (1.67)	71.15 (1.28)	71.92 (0.52)	73.21 (0.67)
	Full Cohort (50750)	82.82 (0.17)	83.04 (0.18)	81.32 (0.18)	77.07 (1.15)	72.72 (1.14)	73.68 (0.56)	75.33 (0.34)
	100	60.44 (8.88)	59.43 (17.91)	57.24 (7.71)	64.09 (42.77)	44.24 (39.98)	50.74 (10.71)	49.12 (28.56)

Bi-GRU+Med-BERT	200	74.44 (3.09)	81.64 (10.38)	71.26 (3.61)	59.09 (28.99)	72.01 (16.31)	69.44 (4.27)	58.15 (24.11)
	300	76 (3.59)	77.19 (8.64)	72.97 (4.21)	66.84 (24.67)	70.03 (13.24)	72.3 (10.12)	64.17 (22.67)
	400	75.85 (3.5)	79.73 (5.3)	73.07 (4.12)	62.58 (33.76)	67.1 (26.57)	68.49 (9.81)	57.27 (29.63)
	500	77.92 (1.04)	79.48 (6.68)	74.99 (0.9)	69.65 (17.45)	70.91 (8.77)	71.15 (3.49)	68.5 (12.69)
	1000	80.14 (0.85)	78.99 (4.03)	77.88 (0.72)	76.57 (2.43)	68.92 (3.37)	71.01 (1.69)	73.64 (0.65)
	2000	81.61 (0.49)	82.02 (2.46)	79.55 (0.71)	74.26 (2)	73.47 (1.95)	73.52 (1)	73.86 (0.71)
	5000	83.12 (0.27)	83.37 (1.54)	81.21 (0.3)	77.17 (1.91)	73.05 (1.59)	73.95 (0.72)	75.51 (0.64)
	10000	83.94 (0.16)	84.15 (1.06)	82.17 (0.16)	78.79 (2.5)	72.78 (2.31)	74.18 (1.05)	76.38 (0.68)
	Full Cohort (50750)	85.39 (0.05)	85.39 (0.07)	83.87 (0.05)	79.51 (2.5)	75.21 (2.53)	76.12 (1.3)	77.74 (0.56)
RETAIN	100	58.75 (8.01)	70.28 (20.26)	56.13 (6.8)	56.67 (27.96)	54.51 (23.81)	55.69 (8.54)	52.95 (14.8)
	200	66.94 (6.04)	75.83 (12.86)	63.27 (6.06)	51.74 (21.84)	69.76 (17.61)	64.55 (6.8)	54.46 (13.97)
	300	74.04 (1.53)	79.36 (8.61)	71.23 (2.23)	46.6 (23.14)	80.09 (13.58)	72.55 (6.01)	52.65 (16.74)
	400	71.88 (4.19)	72.93 (7.49)	68.91 (4.78)	55.42 (22.33)	72.34 (15.15)	67.92 (6.11)	58.04 (14.28)
	500	74.02 (1.13)	79.37 (6.39)	70.96 (1.85)	56.28 (25.6)	73.02 (17.42)	70.51 (6.8)	58 (16.37)
	1000	74.29 (2.21)	76.04 (1.91)	71.59 (2.62)	61.94 (13.33)	72.31 (9.68)	69.64 (4.13)	64.52 (7)
	2000	75.43 (1.44)	76.16 (2.68)	72.88 (1.7)	60.05 (15.83)	74.65 (9.82)	70.98 (3.36)	63.55 (9.58)
	5000	79.47 (0.33)	79.39 (1.29)	77.41 (0.39)	74.75 (2.28)	69.52 (2.28)	70.87 (0.95)	72.73 (0.71)
	10000	80.84 (0.32)	81.27 (1.18)	78.92 (0.36)	75.45 (1.39)	71.29 (1.6)	72.26 (0.77)	73.81 (0.38)
	Full Cohort (50750)	83.28 (0.16)	83.44 (0.25)	81.35 (0.16)	77.2 (0.62)	73.7 (0.63)	74.41 (0.33)	75.77 (0.21)
RETAIN+Med-BERT	100	63.53 (4.91)	80.16 (13.14)	60.44 (5.45)	47.29 (49.01)	56.69 (47.55)	45.35 (25.99)	34.78 (33.34)
	200	70.89 (4.24)	81.44 (9.7)	67.6 (4.58)	62.34 (36.47)	59.18 (30.44)	58.12 (22.43)	54.44 (25.57)
	300	73.49 (6.46)	77.22 (7.27)	70.5 (6.49)	43.83 (35.27)	76.91 (22.42)	71.76 (10.6)	43.93 (29.78)
	400	76.37 (2.96)	78.24 (5.23)	72.99 (3.94)	73.89 (11.69)	63.67 (19.74)	68.31 (6.31)	70.1 (3.43)
	500	76.5 (2.91)	79.55 (5.74)	73.35 (3.51)	41.1 (24.07)	84.79 (13.26)	76.61 (7.25)	48.6 (19.6)
	1000	77.88 (2.85)	79.37 (3.6)	74.03 (3.41)	72.95 (7.46)	68.79 (10.58)	70.5 (4.7)	71.28 (2.26)
	2000	80.55 (1.09)	80.56 (2.06)	77.29 (1.83)	68.87 (6.22)	76.38 (4.51)	74.48 (2.19)	71.34 (2.83)
	5000	82.96 (0.35)	83.41 (1.15)	80.63 (0.59)	76.35 (2.31)	73.97 (2.01)	74.42 (0.94)	75.34 (0.72)
	10000	83.92 (0.16)	84.06 (0.86)	81.95 (0.22)	77.74 (2.56)	73.82 (2.4)	74.67 (1.15)	76.14 (0.67)
	Full Cohort (50750)	85.33 (0.09)	85.36 (0.11)	83.63 (0.11)	78.07 (2.73)	76.09 (2.49)	76.44 (1.26)	77.2 (0.74)
Logistic Regression (LR)*	100	72.49 (2.16)	68.75 (16.44)	70.43 (2.09)	71.57 (9.7)	61.54 (9.25)	65.13 (2.52)	67.78 (3.81)
	200	74.05 (0.44)	76.06 (9.62)	71.6 (0.45)	70.3 (4.29)	65.37 (3.62)	66.85 (1.08)	68.44 (1.63)
	300	75.06 (0.78)	76.01 (8)	72.91 (0.82)	70.69 (3.37)	66.6 (2.97)	67.75 (1.16)	69.13 (1.35)
	400	75.68 (0.76)	74.33 (8.35)	73.4 (1.09)	69.59 (3.57)	68.7 (3.77)	68.86 (1.55)	69.15 (1.22)
	500	76.03 (0.89)	76.21 (6.15)	73.48 (1.06)	72.03 (2.76)	66.84 (3.19)	68.33 (1.49)	70.08 (0.9)
	1000	76.54 (0.68)	75.51 (3.46)	74.06 (0.65)	71.4 (2.06)	68.1 (2.49)	68.95 (1.23)	70.13 (0.88)
	2000	77.15 (0.68)	77.93 (3.63)	74.67 (0.85)	71.4 (2.06)	69.33 (1.36)	69.75 (0.58)	70.55 (0.99)
	5000	78.28 (0.37)	78.4 (2.16)	75.93 (0.44)	72.47 (1.27)	70.29 (0.71)	70.73 (0.35)	71.58 (0.66)
	10000	79 (0.18)	79.33 (0.93)	76.47 (0.23)	74.06 (0.74)	69.84 (0.61)	70.87 (0.31)	72.43 (0.34)

	Full Cohort (50750)	81.01 (0)	81.22 (0)	78.52 (0)	77.12 (0)	70.83 (0)	72.36 (0)	74.66 (0)

B. Experiement 3 - Additional Metrics for PaCa-Cerner prediction evalution using smaller training set size

Threshold used for Sensitivity, Specificity, Precision and F1-score is 0.5

Model	Training Set size	TEST AUC	Validation AUC	AUPRC	Sensitivity	Specificity	Precision (PPV)	F1-score
GRU	100	50.16 (2.88)	73.2 (13.19)	34.47 (3.26)	22.76 (16.48)	80.74 (15.01)	35.63 (3.66)	24.38 (13.91)
	200	56.59 (3.64)	70.43 (10.56)	41.46 (4.39)	42.49 (14.27)	69.23 (13.15)	40.25 (4.61)	39.52 (9.9)
	300	55.64 (3.86)	68.85 (5.42)	40.88 (4.89)	36.48 (15.51)	73.89 (12.78)	40.21 (3.58)	35.9 (11.8)
	400	57.79 (3.06)	67.77 (10.9)	42.56 (3.81)	42.36 (9.67)	70.26 (7.94)	40.39 (3.56)	40.75 (5.47)
	500	58.53 (1.67)	67.04 (6.47)	43.04 (1.88)	43.61 (8.31)	70.02 (8.05)	41.09 (3.02)	41.73 (4.15)
	1000	60.64 (2.07)	69.24 (3.59)	47.2 (1.9)	44.2 (5.59)	72.33 (4.88)	43.09 (2.99)	43.44 (3.24)
	2000	65.17 (1.04)	70.89 (4.31)	53.54 (1.69)	52.45 (4.18)	70.37 (4.55)	45.64 (2.29)	48.66 (1.56)
	5000	70.64 (1.94)	75.55 (2.33)	61.86 (2.28)	52.2 (7.42)	78.61 (4.53)	53.75 (2.23)	52.59 (3.18)
	10000	75.38 (1.28)	79.33 (1.27)	68.21 (1.32)	54.4 (5.91)	83.39 (4.7)	61.31 (4.67)	57.26 (1.94)
	Full Cohort (19250)	78.26 (0.84)	81.63 (0.3)	71.4 (0.9)	58.42 (2.53)	83.54 (2.61)	62.76 (2.99)	60.41 (0.98)
GRU+Med-BERT	100	56.89 (7.3)	73.78 (17.02)	36.65 (5.53)	23.8 (40.9)	76.64 (41.1)	24.95 (19.48)	14.6 (20.13)
	200	66.67 (4.51)	72.71 (8.9)	48.29 (6.68)	13.03 (31.15)	89.49 (31.11)	36.05 (35.04)	9.83 (17.92)
	300	68.74 (5.94)	77.36 (11.94)	52 (7.8)	36.25 (41.26)	73.91 (39.85)	50.07 (34.92)	27.08 (23.76)
	400	71.99 (6.7)	73.58 (7.04)	57.57 (9.93)	47.39 (26.63)	77.5 (28.67)	53.21 (23.31)	45.49 (17.36)
	500	74.19 (1.69)	77.79 (7.43)	61.4 (2.96)	38.51 (22.09)	87.06 (13.06)	68.65 (15.93)	42.68 (17.01)
	1000	76.08 (1.54)	80.11 (3.93)	63.46 (2.8)	52.34 (24.16)	79.71 (11.67)	52.45 (21.28)	49.45 (20.05)
	2000	77.76 (1.55)	79.7 (2.68)	65.51 (2.43)	52.77 (12.82)	83.89 (7.47)	62.4 (6.07)	55.69 (6.15)
	5000	79.86 (0.31)	82.5 (1.91)	69.78 (1.24)	65.88 (3.34)	77.67 (3.14)	58.31 (2.33)	61.75 (0.43)
	10000	81.05 (0.22)	82.92 (0.9)	72.81 (0.51)	66.95 (2.73)	78.31 (2.99)	59.39 (2.3)	62.86 (0.51)
	Full Cohort (19250)	82.13 (0.24)	84.06 (0.21)	74.84 (0.19)	64.52 (1.71)	82.86 (1.63)	63.99 (1.61)	64.22 (0.4)
Bi-GRU	100	51.31 (3.39)	54.54 (11.33)	33.88 (2.3)	41.8 (21.32)	61.12 (21.84)	33.95 (3.15)	35.37 (7.93)
	200	49.78 (4.08)	52.19 (12.94)	32.78 (3.08)	40.44 (23.04)	58.7 (26.69)	33.03 (4.04)	32.65 (9.77)
	300	51.75 (4.33)	60.1 (10.63)	34.74 (4.02)	24.23 (23.16)	78.98 (24.35)	37.52 (7.09)	24.15 (13.48)
	400	54.86 (4.4)	59.55 (11.64)	37.68 (5.29)	42.58 (20.52)	64.1 (23.65)	38.23 (6.39)	37.12 (8.09)
	500	54.1 (5.77)	60.01 (10.33)	37.57 (6.25)	19.83 (18.7)	85.85 (16.72)	42.52 (7.44)	22.26 (15.59)
	1000	65.58 (2.59)	69.59 (5.23)	52.48 (3.45)	46.46 (3.28)	76.44 (4.68)	48.54 (4.67)	47.34 (2.95)

[illegible]	2000	68.44 (1.67)	73.07 (3.63)	57.15 (3.09)	46.93 (8.5)	79.76 (6.1)	52.97 (4.24)	49.04 (4.68)
	5000	72.03 (0.7)	76.16 (2.57)	62.94 (1.1)	52.77 (2.98)	79.65 (2.57)	55.09 (2.04)	53.82 (1.25)
	10000	74.6 (0.63)	77 (0.92)	66.74 (0.67)	56.76 (2.16)	80.07 (1.2)	57.29 (0.81)	57 (0.96)
	Full Cohort (19250)	76.09 (0.61)	79.65 (0.56)	69.05 (0.56)	57.09 (1.67)	82.36 (1.27)	60.41 (1.22)	58.67 (0.74)
Bi-GRU+Med-BERT	100	56.23 (7.13)	75.83 (18.61)	37.31 (6.72)	12.45 (31.06)	88.31 (31.19)	28.32 (33.26)	8.58 (16.26)
	200	66.49 (6.99)	78.29 (9.58)	50.56 (10.61)	9.5 (14.68)	96.34 (7.03)	42.73 (41.03)	12.85 (17.66)
	300	74.17 (2.94)	80.37 (9.27)	61.75 (3.8)	32.64 (18.7)	92.09 (7.44)	73.95 (13.84)	40.4 (17.78)
	400	75.29 (3.15)	78.57 (4.85)	63.06 (4.57)	34.76 (24.46)	90.4 (9.61)	64.98 (26.96)	39.38 (22.95)
	500	75.43 (1.82)	77.77 (7.21)	63.12 (3.89)	45.7 (18.08)	84.81 (11.31)	65.31 (14.59)	49.33 (11.35)
	1000	76.93 (2.51)	80.94 (4)	65.83 (3.68)	45.97 (26.37)	84.96 (10.88)	69.28 (18.32)	46.27 (23.29)
	2000	79.02 (1.06)	80.66 (2.34)	69.39 (1.03)	60.13 (8.07)	80.52 (7.71)	60.77 (7.26)	59.58 (2.06)
	5000	80.92 (0.48)	83.32 (1.55)	72.37 (0.53)	64.38 (2.54)	80.29 (2.72)	60.74 (2.26)	62.43 (0.68)
	10000	81.52 (0.32)	83.35 (0.86)	73.89 (0.25)	63.55 (2.29)	82.63 (2.26)	63.39 (2.24)	63.4 (0.52)
	Full Cohort (19250)	82.23 (0.29)	84.32 (0.13)	75.08 (0.36)	63.82 (2.75)	83.59 (2.81)	64.89 (2.86)	64.25 (0.69)
RETAIN	100	51.91 (4.01)	63.7 (21.91)	35.79 (3.3)	49.97 (8.07)	52.57 (5.29)	33.03 (2.55)	39.64 (4.24)
	200	51.36 (6.91)	55.23 (6.35)	35.52 (4.81)	41.52 (18.56)	62.78 (14.49)	34.55 (4.94)	36.14 (8.55)
	300	55.5 (5.12)	61.78 (13.5)	40.48 (5.85)	45.49 (13.47)	63.99 (14.95)	38.63 (5.81)	40.33 (5.72)
	400	55.62 (7.58)	66.65 (9.5)	40.74 (6.7)	27.77 (15.33)	83.06 (10.29)	44.35 (8.79)	32.23 (10.35)
	500	57.58 (9.69)	64.82 (11.11)	42.22 (8.1)	39.1 (15.49)	73.58 (13.64)	42.42 (9.82)	39.1 (10.57)
	1000	66.03 (4.87)	71.89 (4.48)	53.8 (6.15)	38.09 (9.63)	85.79 (5.56)	56.44 (5.83)	44.68 (7.02)
	2000	71.24 (3.78)	73.45 (3.28)	61.61 (4.36)	41.4 (5.86)	88.32 (2.45)	62.67 (3.48)	49.6 (4.81)
	5000	76.5 (0.48)	78.73 (1.6)	68.59 (0.69)	49.28 (3.17)	88.03 (2.4)	66.25 (3.07)	56.38 (1.37)
	10000	78.43 (0.41)	79.87 (0.69)	70.77 (0.39)	50.76 (2.28)	89.05 (1.49)	68.7 (2.12)	58.31 (0.99)
	Full Cohort (19250)	79.68 (0.32)	80.99 (0.32)	72.02 (0.3)	52.78 (1.76)	88.35 (0.79)	68.1 (1.03)	59.45 (1.04)
RETAIN+Med-BERT	100	58.49 (9.49)	74.02 (12.18)	39.89 (9.18)	42.82 (42.12)	62.22 (39.31)	26.74 (15.61)	28.03 (22.95)
	200	59.58 (8.6)	71.51 (12.05)	40.49 (7.87)	4.13 (8.62)	97.75 (4.93)	30.31 (35.37)	5.77 (11.6)
	300	60.61 (8.26)	71.82 (12.36)	42.78 (9.97)	10.33 (19.24)	92.61 (14.3)	44.82 (40.39)	10.52 (16.85)
	400	69.3 (6.45)	78.19 (8.32)	53.11 (8.22)	6.09 (17.35)	98.25 (5.43)	33.19 (44.03)	6.84 (17.95)
	500	74.01 (2.6)	79.56 (6.57)	59.56 (3.64)	14.1 (19.42)	96.61 (6.85)	57.46 (41.8)	18.38 (20.93)
	1000	76.35 (1.21)	79.61 (3.59)	63.92 (2.28)	20.49 (17.97)	96.13 (7.29)	85.52 (14.21)	28.16 (18.37)
	2000	78.12 (1.18)	80.54 (1.99)	65.74 (2.77)	33.69 (14.33)	93.59 (7.41)	77.44 (11.28)	44.05 (11.49)
	5000	79.7 (0.62)	82.46 (1.29)	68.25 (1.27)	35.12 (8.17)	95.41 (2.43)	79.69 (5.43)	47.92 (6.91)
	10000	80.36 (0.32)	83.08 (0.99)	69.43 (1.46)	41.82 (5.7)	93.96 (2.14)	77.24 (4.36)	53.84 (4.2)

	Full Cohort (19250)	81.3 (0.55)	83.34 (0.13)	71.78 (3.1)	59.24 (9.26)	84.94 (6.43)	66.36 (6.51)	61.71 (2.43)
Logistic Regression (LR)*	100	64.93 (3.83)	76.39 (16.16)	53.37 (3.28)	35.43 (7.86)	87.62 (7.8)	60.78 (10.26)	43.41 (4.3)
	200	70.68 (2.2)	71.35 (10.96)	59.69 (2.01)	45.3 (6.91)	87.18 (3.47)	62.94 (3.45)	52.24 (3.94)
	300	70.59 (1.56)	71.78 (8.16)	60.71 (1.59)	44.89 (4.4)	87.68 (3.29)	63.71 (4.35)	52.39 (2.29)
	400	71.55 (1.43)	71.02 (5.44)	61.85 (1.46)	46.48 (3.65)	87.99 (3.01)	65.04 (4.02)	53.99 (1.33)
	500	71.47 (1.69)	69.66 (5.23)	61.85 (1.51)	46.59 (3.44)	87.62 (3.3)	64.44 (4.48)	53.85 (1.47)
	1000	73.7 (0.81)	75.23 (3.78)	65.02 (0.73)	51.32 (3.04)	87.01 (2.26)	65.26 (2.74)	57.33 (1.01)
	2000	75.04 (1.16)	75.51 (2.76)	67.33 (1.21)	52.66 (1.31)	87.66 (1.12)	66.81 (2)	58.88 (1.19)
	5000	76.91 (0.6)	77.72 (2.59)	69.99 (0.45)	54.62 (1.29)	88.06 (0.84)	68.31 (1.15)	60.69 (0.69)
	10000	78.63 (0.3)	78.72 (1.01)	72.18 (0.26)	55.49 (0.94)	89.21 (0.59)	70.78 (0.91)	62.2 (0.48)
	Full Cohort (19250)	79.94 (0)	79.45 (0)	73.59 (0)	56.82 (0)	89.49 (0)	71.79 (0)	63.43 (0)

C. Experiement 3 - Additional Metrics for PaCa-Truven prediction evalution using smaller training set size

Threshold used for Sensitivity, Specificity, Precision and F1-score is 0.5

Model	Training Set size	TEST AUC	Validation AUC	AUPRC	Sensitivity	Specificity	Precision (PPV)	F1-score
GRU	100	55.23 (4.18)	73.49 (15.84)	38.39 (4.07)	26.27 (5.88)	79.69 (4.48)	39.79 (4.44)	31.39 (5.19)
	200	59.04 (3.76)	69.82 (6.92)	42.00 (3.78)	33.52 (12.19)	78.09 (6.81)	42.99 (3.74)	36.65 (10.55)
	300	61.33 (3.79)	71.51 (10.05)	44.16 (4.17)	34.47 (13.24)	79.93 (7.37)	46.11 (3.07)	38.12 (11.19)
	400	62.43 (3.26)	71.22 (10.08)	45.80 (3.54)	37.04 (7.83)	79.33 (4.89)	47.88 (3.99)	41.33 (5.52)
	500	65.24 (1.64)	68.51 (7.23)	50.05 (2.37)	40.91 (3.97)	79.30 (4.90)	50.7 (3.67)	45.00 (1.32)
	1000	67.32 (1.06)	69.98 (3.55)	52.65 (1.83)	43.43 (7.27)	79.73 (6.00)	52.72 (2.88)	47.15 (3.19)
	2000	70.45 (1.07)	69.56 (3.38)	57.12 (1.52)	47.44 (7.99)	79.76 (5.98)	55.11 (3.79)	50.38 (3.61)
	5000	73.15 (1.50)	72.81 (2.87)	62.16 (1.61)	46.61 (7.15)	84.17 (5.49)	61.04 (5.31)	52.2 (3.45)
	10000	75.95 (0.54)	74.41 (2.12)	65.53 (0.74)	45.58 (3.67)	88.08 (2.04)	66.29 (2.39)	53.87 (2.13)
	Full Cohort (23609)	78.17 (0.21)	77.31 (0.35)	68.93 (0.19)	49.54 (4.02)	88.06 (2.67)	68.2 (2.92)	57.2 (1.61)
+Med-BERT	100	53.72 (5.74)	61.24 (21.96)	36.85 (4.00)	49.76 (52.06)	50.57 (51.8)	24.41 (17.23)	25.63 (26.23)
	200	59.88 (6.13)	71.19 (13.57)	42.5 (6.08)	20.89 (41.57)	79.77 (41.76)	20.79 (24.2)	11.80 (20.95)
	300	63.49 (7.62)	73.48 (11.09)	46.18 (7.97)	10.9 (30.78)	89.42 (31.12)	27.41 (35.66)	6.86 (15.76)
	400	66.2 (6.15)	73.58 (6.13)	49.53 (8.56)	17.24 (22.9)	93.11 (10.54)	42.30 (33.99)	20.00 (23.31)
	500	69.06 (8.53)	75.85 (10.5)	55.47 (9.56)	16.47 (22.38)	94.29 (10.29)	54.48 (33.29)	19.84 (21.38)
	1000	73.81 (0.92)	73.21 (4.71)	61.35 (1.83)	21.37 (21.54)	94.09 (8.77)	54.53 (32.94)	26.81 (22.6)

GRU	2000	75.75 (0.72)	75.51 (3.04)	64.32 (1.39)	20.15 (15.68)	96.53 (4.03)	83.46 (11.69)	28.71 (19.02)
	5000	77.9 (0.35)	76.7 (2.39)	67.75 (0.64)	40.41 (11.99)	91.48 (4.56)	73.15 (7.47)	50.31 (9.72)
	10000	79.1 (0.24)	77.65 (1.68)	69.43 (0.49)	52.73 (4.68)	87.09 (2.77)	67.86 (2.82)	59.13 (2.02)
	Full Cohort (23609)	80.37 (0.12)	79.33 (0.17)	71.21 (0.28)	56.81 (2.00)	86.07 (1.32)	67.56 (1.31)	61.68 (0.66)
Bi-GRU	100	50.45 (1.94)	59.66 (17.95)	34.23 (1.32)	37.54 (21.46)	62.66 (23.22)	34.65 (1.8)	32.97 (9.44)
	200	50.43 (1.59)	51.92 (15.51)	34.19 (1.12)	28.1 (21.16)	72.21 (21.78)	34.74 (1.91)	27.44 (10.94)
	300	52.6 (2.02)	55.37 (11.29)	35.78 (1.64)	22.88 (12.28)	79.02 (12.34)	36.72 (2.82)	26.2 (8.54)
	400	55.05 (4.58)	58.98 (10.97)	37.94 (3.90)	19.22 (13.51)	83.99 (14.00)	40.2 (6.02)	23.33 (9.85)
	500	53.46 (5.97)	58.58 (8.37)	36.83 (5.14)	37.72 (22.29)	64.99 (25.26)	38.61 (7.72)	33.66 (10.3)
	1000	62.15 (7.57)	64.33 (9.19)	46.09 (7.94)	34.61 (16.91)	78.88 (19.14)	48.57 (9.66)	37.78 (10.80)
	2000	70.18 (0.99)	69.87 (3.19)	55.6 (1.39)	41.12 (4.45)	84.21 (3.05)	57.32 (2.64)	47.65 (2.64)
	5000	73.36 (0.50)	73.12 (2.56)	60.95 (0.76)	41.75 (1.89)	87.38 (1.17)	62.82 (1.41)	50.12 (1.16)
	10000	74.74 (0.50)	74.29 (1.69)	63.64 (0.70)	44.96 (2.12)	87.2 (0.86)	64.16 (0.87)	52.84 (1.40)
	Full Cohort (23609)	76.79 (0.29)	76.66 (0.21)	66.87 (0.32)	46.65 (0.81)	88.61 (0.63)	67.61 (1.05)	55.2 (0.58)
Bi-GRU+Med-BERT	100	54.57 (7.05)	56.85 (18.97)	37.58 (5.96)	20.9 (34.83)	78.47 (35.71)	22.74 (20.78)	14.5 (20.11)
	200	59.54 (4.48)	63.22 (14.21)	41.62 (3.61)	0.88 (2.78)	99.54 (1.47)	4.91 (15.54)	1.49 (4.71)
	300	67.78 (4.43)	75.3 (8.9)	51.37 (5.42)	13.02 (20.18)	95.33 (7.93)	29.88 (32.71)	15.49 (22.68)
	400	67.54 (5.4)	73.05 (8.54)	51.02 (7.00)	12.24 (18.47)	95.78 (7.31)	43.37 (39.43)	15.33 (20.86)
	500	70.7 (4.49)	75.78 (8.35)	56.25 (6.32)	26.65 (22.73)	90.8 (9.85)	63.59 (26.98)	31.18 (22.98)
	1000	74.03 (1.22)	74.16 (4.73)	60.89 (2.26)	12.1 (10.1)	98.27 (1.78)	65.38 (35.03)	19.38 (15.2)
	2000	76.07 (0.47)	75.9 (3.42)	64.04 (1.09)	33.77 (15.03)	92.2 (5.29)	72.59 (8.26)	43.19 (14.5)
	5000	77.94 (0.25)	76.92 (2.26)	67.23 (0.62)	47.16 (5.6)	88.4 (2.89)	67.92 (3.29)	55.33 (3.17)
	10000	79.25 (0.22)	77.88 (1.72)	69.42 (0.81)	56.75 (3.36)	84.56 (2.17)	65.33 (1.9)	60.64 (1.19)
	Full Cohort (23609)	80.57 (0.21)	79.45 (0.22)	71.54 (0.45)	56.8 (1.5)	86.02 (0.96)	67.45 (0.99)	61.65 (0.54)
RETAIN	100	51.73 (3.01)	57.5 (19.24)	34.78 (2.08)	45.47 (11.36)	57.19 (10.29)	35.15 (2.35)	39.13 (4.73)
	200	54.57 (4.3)	61.01 (15)	37.61 (3.37)	17.43 (11.76)	85.71 (14.16)	42.05 (6.33)	22 (9.60)
	300	54.14 (3.47)	57.1 (10.46)	37.58 (3.22)	17.82 (15.40)	85.3 (16.33)	43.92 (7.97)	21.22 (11.3)
	400	53.88 (6.08)	59.6 (9.87)	37.07 (5.46)	25.99 (13.39)	76.94 (14.96)	39.78 (9.64)	28.3 (11.29)
	500	57.65 (4.69)	67.11 (7.61)	40.42 (4.27)	17.1 (17.65)	87.22 (16.85)	47.75 (9.84)	20.12 (12.92)
	1000	57.91 (6.54)	63.56 (7.1)	41.6 (6.88)	16.67 (13.04)	89.49 (12.34)	50.36 (12.54)	21.85 (12.95)
	2000	69 (2.72)	69.37 (4.18)	54.04 (3.31)	27.32 (10.28)	91.76 (4.01)	63.68 (3.31)	36.88 (11.02)
	5000	73.59 (0.68)	74.32 (1.63)	61.07 (1.38)	38.62 (2.20)	89.94 (0.68)	66.19 (0.82)	48.75 (1.8)
	10000	75.75 (0.46)	75.77 (0.87)	64.96 (0.6)	42.3 (0.92)	89.84 (0.68)	67.99 (1.35)	52.14 (0.77)

	Full Cohort (23609)	78.02 (0.19)	77.8 (0.20)	68.93 (0.35)	45.74 (0.63)	90.24 (0.54)	70.5 (0.96)	55.48 (0.40)
RETAIN+Med-BERT	100	55.72 (3.99)	68.33 (8.82)	38.61 (2.99)	26.96 (43.41)	73.78 (42.37)	18.61 (19.8)	15.24 (23.62)
	200	59.63 (7.48)	71.06 (11.55)	42.84 (7.60)	15.59 (33.72)	84.51 (33.86)	17.42 (24.06)	9.91 (19.05)
	300	61.36 (7.58)	69.63 (9.67)	46.38 (8.38)	6.55 (20.24)	93.18 (21.34)	22.4 (33.70)	4.58 (13.59)
	400	67.47 (6.21)	72.17 (6.93)	52.37 (8.11)	3.59 (11.24)	96.93 (9.67)	27.05 (41.06)	3.71 (11.48)
	500	67.39 (6.23)	75.13 (3.97)	52.05 (7.60)	4.9 (8.76)	98.69 (2.71)	36.52 (40.45)	7.67 (12.74)
	1000	71.26 (4.87)	71.55 (5.03)	57.69 (6.84)	7.71 (9.43)	98.07 (3.78)	48.48 (44.4)	12.18 (14.36)
	2000	75.67 (0.73)	75.23 (3.59)	63.46 (1.51)	0.49 (1.07)	100 (0)	30 (48.30)	0.96 (2.07)
	5000	77.91 (0.25)	77.3 (2.11)	67.27 (0.42)	9.23 (9.25)	99.38 (1.27)	94.66 (6.4)	15.46 (13.67)
	10000	78.85 (0.24)	77.45 (1.28)	68.59 (0.48)	17.2 (7.57)	98.71 (1.18)	89.82 (6.02)	27.98 (10.24)
	Full Cohort (23609)	79.98 (0.17)	79.2 (0.16)	69.39 (0.97)	33.43 (8.33)	95.49 (2.83)	80.56 (5.16)	46.4 (7.02)
Logistic Regression (LR)*	100	67.44 (2.77)	67.28 (13.72)	51.77 (2.96)	25.99 (9.85)	90.43 (5.27)	59.31 (4.53)	34.9 (8.58)
	200	69.74 (1.15)	73.98 (13.98)	54.83 (1.55)	33.08 (9.3)	88.59 (4.48)	60.63 (3.92)	41.75 (8.16)
	300	70.98 (1.16)	70.91 (6.95)	56.66 (1.61)	36.56 (3.57)	87.9 (2.22)	60.85 (2.74)	45.52 (2.5)
	400	70.56 (1.42)	72.32 (6.97)	56.08 (1.36)	39.36 (5.46)	85.99 (3.37)	59.26 (2.99)	47 (3.10)
	500	71.28 (1.36)	74.45 (3.31)	57.4 (1.77)	40.94 (3.88)	86.19 (2.17)	60.34 (2.48)	48.64 (2.5)
	1000	71.98 (0.86)	72.79 (3.97)	58.84 (1.20)	43.25 (2.92)	85.59 (1.62)	60.53 (1.48)	50.37 (1.73)
	2000	73.38 (0.56)	72.55 (3.1)	61.36 (0.61)	46.67 (2.09)	85.21 (0.97)	61.67 (0.66)	53.1 (1.24)
	5000	74.58 (0.51)	74.26 (1.71)	63.69 (0.51)	46.02 (0.62)	87.04 (0.58)	64.4 (1.10)	53.68 (0.65)
	10000	75.72 (0.43)	75.5 (1.13)	65.33 (0.46)	46.23 (1.12)	87.81 (0.58)	65.9 (0.90)	54.33 (0.81)
	Full Cohort (23609)	77.28 (0)	77.11 (0)	67.33 (0)	45.52 (0)	89.17 (0)	68.16 (0)	54.58 (0)
